# Utility of binding protein fusions to immunoglobulin heavy chain constant regions from mammalian and avian species

**DOI:** 10.1016/j.jbc.2025.108324

**Published:** 2025-02-18

**Authors:** Ningyu Zhu, Philip M. Smallwood, John Williams, Yanshu Wang, Jeremy H. Nathans

**Affiliations:** 1 Department of Molecular Biology and Genetics, Johns Hopkins University School of Medicine, Baltimore, Maryland, USA; 2 Howard Hughes Medical Institute, Johns Hopkins University School of Medicine, Baltimore, Maryland, USA; 3 Department of Neuroscience, Johns Hopkins University School of Medicine, Baltimore, Maryland, USA; 4 Department of Ophthalmology, Johns Hopkins University School of Medicine, Baltimore, Maryland, USA

**Keywords:** immunoglobulin G, immunohistochemistry, fusion protein, toxin, chemokine, DARPin

## Abstract

Antibodies are of central importance as reagents for the localization of proteins and other biomolecules in cells and tissues. To expand the repertoire of antibody-based reagents, we have constructed a series of plasmid vectors that permit expression of amino-terminal fusions to the hinge and Fc regions from goat, guinea pig, human, mouse, and rabbit immunoglobulin Gs, and chicken immunogloblin Y. The resulting fusion proteins can be produced in transfected mammalian cells and detected with commercially available and species-specific secondary antibody reagents. We demonstrate the utility of this platform by constructing and testing Fc fusions with DARPin, single-chain Fv, nanobody, toxin, and chemokine partners. The resulting fusion proteins were used to detect their targets in tissue sections or on the surface of transfected cells by immunofluorescent staining or on the surface of immune cells by flow cytometry. By expanding the range of Fc sequences available for fusion protein production, this platform will expand the repertoire of primary antibody reagents for multiplexed immunostaining and fluorescence-activated cell sorting analyses.

Over the past 50 years, antibody-based localization of proteins and other biomolecules has become a foundational technique for characterizing cells and tissues in biomedical research and clinical diagnostics (1, 2). The availability of primary antibodies from different species, together with species-specific secondary anti-immunoglobulin G (IgG) antibodies that are conjugated to fluorophores or enzymes, permits the detection of target molecules by immunofluorescence, immunohistochemistry, or flow cytometry. The production of well-validated primary and secondary antibodies has also synergized with advances in instrumentation for light microscopy and flow cytometry.

The first generation of antibody reagents consisted of polyclonal antisera generated in species such as rabbit and goat. The second generation consisted of monoclonal antibodies (mAbs) produced by hybridomas that were generated from mice or rats and subsequently from rabbits and humans. The most recent approach to generating antibody reagents involves sequencing complementary DNA copies of mRNAs from individual plasma cells to determine the amino acid sequences of the complementarity-determining regions of the heavy and light chains, followed by production of the desired Ig as a recombinant protein (3).

Technical challenges associated with the *in vivo* antibody repertoire have driven the development of technologies for the selection of antibodies and other binding proteins *ex vivo* (4, 5, 6, 7). These technologies include phage display, yeast display, and ribosome display, and they have been applied to both Ig and non-Ig protein scaffolds (8, 9, 10, 11). *Ex vivo* display technologies are generally used in conjunction with monomeric protein scaffolds. Thus, for display of dimeric Ig-binding domains, the heavy and light chain variable regions are joined by a flexible linker, effectively creating a monomeric protein, single-chain (sc)Fv, that can be expressed from a single open reading frame. Importantly, the amino acid sequences of binding proteins generated by genetic engineering can be inexpensively preserved and disseminated. The availability of these sequences enables the ready construction of fusion proteins with different partners and the production of these fusions in an appropriate expression system.

The purpose of the present study is to add to the binding protein “tool-kit” by generating, testing, and making available to the scientific community a series of plasmid vectors for expressing binding domains as fusions to dimeric heavy chain constant regions from species for which there are high-quality secondary antibody reagents. As one example, an anti-GFP designed ankyrin repeat protein (DARPin) was produced as a fusion to Fc proteins from six species and then used for immunostaining. Additional Fc fusions were based on scFv, nanobody (NB) (single-domain antibody), protein toxins, and chemokines, all of which bound their targets in tissue sections or on cells. As the field moves increasingly toward recombinant DNA–based production of binding proteins, the ability to create different Fc fusions will increase the utility of each binding protein because it will permit that protein to be used in combination with a wider variety of secondary antibody reagents and with target tissues or cells from a wider variety of species.

## Results

### Plasmid vectors for the production of Fc fusion proteins

**Figure 1:**
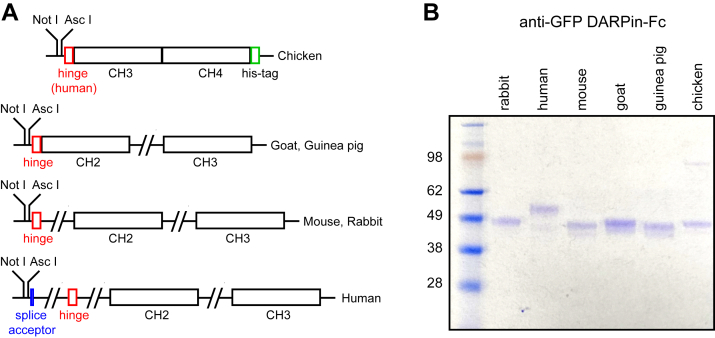
**Design and expression of Fc fusion vectors.***A*, schematic of the Fc expression vectors. Exons (*open rectangles*) and introns (*connecting lines*). *B*, anti-GFP DARPin Fc fusion proteins purified from 0.5 ml of SFCM following transient transfection of 293T cells. The mammalian Fc fusion proteins were captured with protein-G magnetic beads, and the chicken Fc fusion protein was captured with nickel–NTA magnetic beads, resolved by SDS-PAGE, and visualized by Coomassie Blue staining. The mass (in kilodalton) of each of the molecular weight markers is listed to the *left*. NTA, nitrilotriacetic acid; SFCM, serum-free conditioned medium.

To produce secreted Fc fusion proteins from transfected mammalian cells, a set of six plasmid vectors were constructed based on pRK5, a mammalian expression plasmid with an SV40 origin of DNA replication and a cytomegalovirus immediate early enhancer and promoter followed by an intron in the 5′ untranslated region (https://www.addgene.org/vector-database/3944/). The six plasmids contain unique NotI and AscI restriction sites for insertion of the signal peptide and protein-binding domain, followed by a short linker, the IgG heavy chain hinge region, and the constant heavy (CH)2 and CH3 domains that comprise the Fc region from goat, guinea pig, human, mouse, or rabbit IgG (Figs. 1*A* and S1–S5). For chicken IgY, which contains three rather than two CH domains in the Fc region, only the last two CH domains (CH3 and CH4) were included (Figs. 1*A* and S6). IgG and IgY sequences were PCR amplified from genomic DNA, and, for some constructs, some or all the introns were eliminated if they were deemed to be inconveniently large or were of unknown size. Since chicken Fc domains do not bind to protein-A or protein-G, an 8xHis tag was appended to chicken Fc to permit purification by nickel–nitrilotriacetic acid capture.

DNA sequences coding for a signal peptide from human Frizzled (Fzd) 8 joined to a high-affinity anti-GFP DARPin, 3G86.32 (<5 nM affinity; (12)), were inserted between NotI and AscI in each of the six Fc expression plasmids, and the resulting plasmids were transiently transfected into human embryonic kidney 293T (HEK293T) cells. Secreted anti-GFP-DARPin-Fc proteins in serum-free conditioned medium (SFCM) were captured with protein-G magnetic beads (for mammalian Fc fusions) or with nickel–nitrilotriacetic acid (for the chicken Fc fusion) (Fig. 1*B*). Following 48 h of culture, the yield for each of the Fc fusions in SFCM was approximately 0.5 μg/ml. As the 10 ml of SFCM obtained from a single 10 cm plate provides sufficient Fc fusion protein for immunostaining of many thousands of slides (at a working dilution of 1:50), we have not attempted to optimize the efficiency of protein production. The six anti-GFP DARPin-Fc fusion plasmids have been deposited at Addgene (ID numbers: 231044-231049).

### Anti-GFP-DARPin fusions to Fc from multiple species

**Figure 2:**
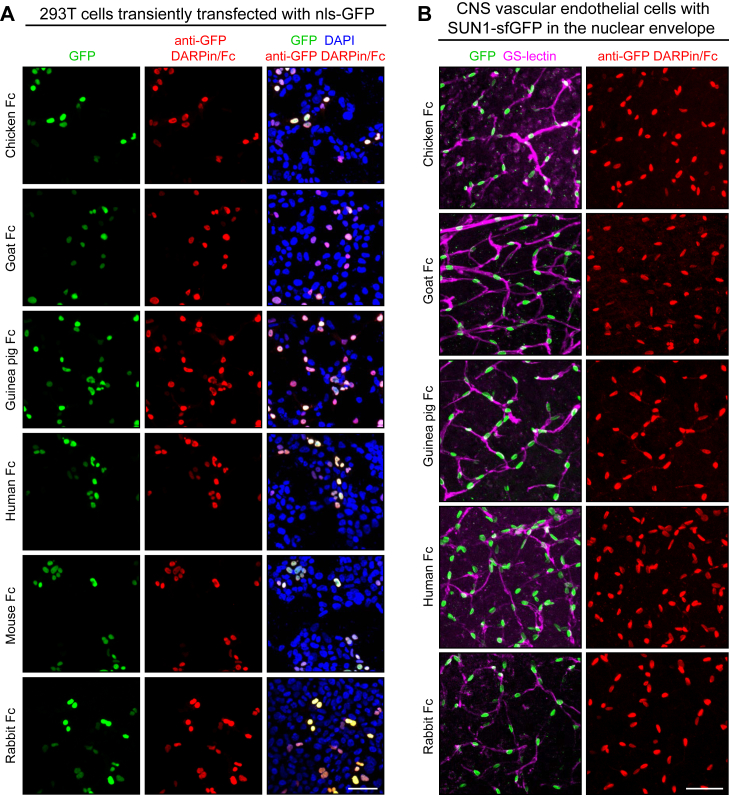
**Immunostaining with anti-GFP DARPin Fc fusions.***A*, 293T cells were transiently transfected with a vector coding for a nuclear-localized GFP, and 2 days later, the cells were fixed, permeabilized, and immunostained with each of the indicated anti-GFP DARPin Fc proteins. *B*, vibratome sections of brain from mice expressing CreER in vascular endothelial cells in the presence of a *LoxP-stop-LoxP* (*LSL*) reporter at the *Rosa26* (*R26*) locus that expresses a SUN1-super-folder (sf) GFP dimer following Cre-mediated recombination (genotype: *VECad-CreER;R26-LSL-SUN1-sfGFP*). Sections were immunostained with GS-lectin, which binds to vascular endothelial cells, together with each of the indicated anti-GFP DARPin Fc proteins. For both (*A*) and (*B*), GFP fluorescence is shown in the *first column*, and immunostaining with anti-GFP DARPin Fc fusion proteins is shown in the *second column*. For these and all other experiments, Fc proteins were produced in transiently transfected 293T cells, collected in serum-free conditioned medium (SFCM), and used at 1:50. Scale bars represent 50 μm.

GFP is widely used as a reporter, and in many applications, its presence is detected with antibodies rather than *via* its native fluorescence (*e.g.*, in paraformaldehyde [PFA] fixed tissues). In addition, superfolder GFP (sfGFP), which contains 12 amino acid substitutions relative to WT GFP (13), is also in widespread use (*e.g.*, (14)). Thus, Fc fusions from diverse species that recognize GFP and its superfolder variant should be of general utility. Immunostaining of HEK293T cells expressing nuclear-localized enhanced GFP show colocalization of endogenous GFP fluorescence and anti-GFP-DARPin-Fc immunostaining for all six Fc species tested (Fig. 2*A*). Similarly, colocalization of endogenous GFP fluorescence and anti-GFP-DARPin-Fc immunostaining was observed in vibratome sections of brain from adult *VECad-CreER;R26-LSL-SUN1-sfGFP* mice expressing SUN1, a nuclear membrane protein, fused to two copies of sfGFP followed by six tandem myc epitopes (Fig. 2*B*). In these mice, the SUN1–sfGFP fusion is expressed specifically in vascular endothelial cells. Tissue immunostaining was not performed with the anti-GFP-DARPin mouse Fc protein because the presence of endogenous mouse IgG generates a high background by binding to antimouse secondary antibodies.

### Tissue staining with scFv, NB, and toxin Fc fusions

To explore the utility of additional classes of binding proteins, Fc fusions were constructed with scFv, NB, toxin, and chemokine partners. We chose protein toxins and chemokines because they typically have multiple disulfide bonds, are highly stable, and bind their cell surface receptors with high affinity. In examining more than 50 fusion partners, we observed that ∼50% of the Fc fusion proteins were secreted from transiently transfected 293T cells at levels >100 ng/ml in SFCM.

**Figure 3:**
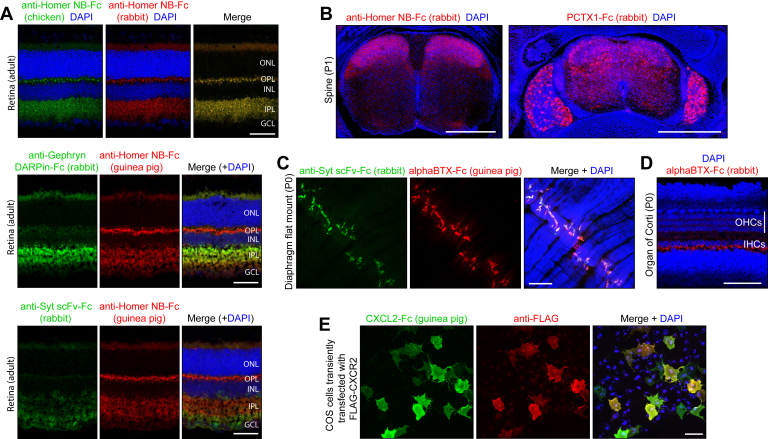
**Immunostaining of mouse tissues with Fc fusions that bind to neuronal proteins.***A*, immunostaining of adult mouse retina with the indicated combinations of Fc fusion proteins. The possibility of crossreactivity between the anti-chicken and anti-rabbit secondary antibodies is ruled out by control experiments such as the one shown in Fig. S2 from Ref. (48), in which these secondary antibodies are used for tissue immunostaining against targets with distinct cellular localizations. *B*, immunostaining of transverse sections of P1 spinal columns with anti-Homer NB-Fc (*left*) and PcTx1-Fc (*right*). The *left image* shows only the spinal cord. The *right image* shows the spinal cord and the flanking DRGs. Dorsal-to-ventral corresponds to *top-to-bottom* in each image. *C*, flatmount of a P1 diaphragm immunostained with anti-Syt scFv-Fc and alphaBTX-Fc. *D*, flatmount of a P1 cochlea immunostained with alphaBTX-Fc. *E*, immunostaining of COS cells transiently transfected with N-terminally FLAG-tagged CXCR2 and immunostained with anti-FLAG monoclonal antibody and CXCL2-Fc. Scale bars in (*A*) and (*D*) represent 50 μm; in (*B*), 500 μm; and in (*C*) and (*E*), 100 μm. alphaBTX, alpha-bungarotoxin; DRG, dorsal root ganglion; GCL, ganglion cell layer; IHC, inner hair cell; INL, inner nuclear layer; IPL, inner plexiform layer; NB, nanobody; OHC, outer hair cell; ONL, outer nuclear layer; OPL, outer plexiform layer; Syt, synaptotagmin.

Homer, Gephryn, and Synaptotagmin are synapse-associated proteins that are widely used as markers for glutamatergic synapses (postsynaptic structures; Homer), inhibitory synapses (postsynaptic structures; Gephryn), and all synapses (presynaptic structures; Synaptotagmin) (15, 16, 17). An anti-Homer1 NB (18), an anti-Gephryn DARPin (19), and an anti-Synaptotagmin-6 scFv (20) were chosen as Fc fusion partners. Immunostaining of adult mouse retina with anti-Homer NB-Fc fusions to chicken Fc and to rabbit Fc shows identical patterns of immunostaining, with signal localized to the two synaptic layers, the inner plexiform layer (IPL) and the outer plexiform layer (OPL) (Fig. 3*A*, *top panels*). Immunostaining of adult mouse retina with anti-Homer NB-Fc (guinea pig) in combination with anti-Gephryn DARPin-Fc (rabbit) shows a strong anti-Gephryn signal in the IPL and a little or no signal in the OPL, consistent with the presence of large numbers of inhibitory amacrine cell synapses in the IPL but little or no inhibitory signaling in the OPL ((21, 22); Fig. 3*A*, *center panels*). Similar immunostaining of adult mouse retina with anti-Homer NB-Fc (guinea pig) and anti-Synaptotagmin scFv-Fc (rabbit) shows the Synaptotagmin signal in the IPL, OPL, and the ganglion cell layer, the latter presumably reflecting a Synaptotagmin pool destined for axonal transport to presynaptic retinal ganglion cell terminals in the brain ((23); Fig. 3*A*, *bottom panels*). Control immunostaining of mouse brain, retina, and spinal cord sections using only the fluorescent secondary anti-chicken, anti-goat, anti-guinea pig, antihuman, anti-mouse, and anti-rabbit antibodies showed no detectable staining with the confocal microscope settings used here.

Immunostaining of postnatal day (P)1 mouse spine with anti-Homer NB-Fc shows the strongest signal in the dorsal laminae of the spinal cord, where axons from dorsal root ganglion sensory neurons synapse onto second-order neurons (Fig. 3*B*, *left panel*; (24)). Transverse sections of P1 mouse spine were also stained with a Psalmotoxin (PcTx1)–Fc fusion (Fig. 3*B*, *right panel*). PcTx1 is a 40 amino acid cysteine-knot peptide from the venom of the South American tarantula *Psalmopoeus cambridgei*, and it binds to and desensitizes acid-sensing ion channel 1 (ASIC1; (25, 26, 27)). ASIC1 is expressed in a subset of dorsal root ganglion sensory neurons and in a variety of neurons throughout the central nervous system (28). The immunostaining pattern with the PcTx1–Fc fusion is consistent with this pattern of ASIC1 expression.

Alpha-bungarotoxin (BTX), a 74 amino acid peptide with five disulfide bonds, is a high-affinity antagonist of nicotinic acetylcholine receptors (NAChRs) (29). It is derived from the venom of the Taiwanese banded krait snake (*Bungarus multicinctus*) and is widely used as a reagent for visualizing postsynaptic NAChRs at the neuromuscular junction and in central synapses. Immunostaining of flatmounts of neonatal mouse diaphragm with alphaBTX-Fc (guinea pig) and anti-Synaptotagmin scFv-Fc (rabbit) highlights the neuromuscular junctions at the centers of muscle fibers (Fig. 3*C*). AlphaBTX-Fc (rabbit) was also used to visualize NAchRs in the P1 mouse cochlea (Fig. 3*D*). Prior to the onset of hearing, inner hair cells in the neonatal rodent cochlea transiently expresses NAChRs containing the alpha9 subunit, as determined electrophysiologically (30, 31). The binding of alphaBTX-Fc to the inner face of inner hair cells, the site of synaptic contacts from efferent fibers, is consistent with these electrophysiological data.

### Staining cells for flow cytometry with chemokine-Fc fusions

**Figure 4:**
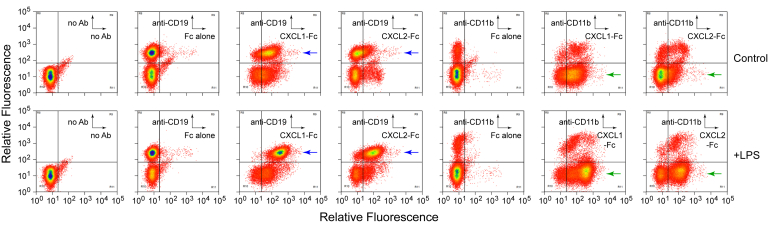
**Flow cytometry of immune cells stained with cytokine-Fc fusion proteins.** Cells were harvested from an adult mouse spleen and immunostained as described in the Experimental procedures section. *Upper row*, splenocytes from a control mouse. *Lower row*, splenocytes from a mouse that received 0.2 mg LPS by IP injection 24 h prior to analysis. For each plot, the sample was gated for live cells and singlets. The primary antibody probes used for each panel are indicated in the *insets*, which show the *X*- and *Y*-axes for each panel. First pair of panels, controls with no primary antibody. Second and fifth pairs of panels, controls with anti-CD19 and anti-CD11b, respectively, on the *Y*-axis, and Fc (rabbit) without a fusion protein partner on the *X*-axis. Third, fourth, sixth, and seventh pairs of panels, experimental panels with all pairwise combinations of anti-CD19 and anti-CD11b on the *Y*-axis and CXCL1Fc (rabbit) and CXCL2-Fc (rabbit) on the *X*-axis. Phycoerythrin (PE) eFluor 576–conjugated donkey anti-rabbit antibodies were used to detect CXCL1-Fc and CXCL2-Fc fusion proteins (*X*-axis). The signals from allophycocyanin (APC) eFluor 780–conjugated anti-CD marker antibodies are displayed on the *Y*-axis. IP, intraperitoneal; LPS, lipopolysaccharide.

Current phenotyping of immune cells by flow cytometry relies on a collection of mAbs to more than 350 cell surface markers, each designated by a cluster of differentiation (CD) number (https://www.bdbiosciences.com/content/dam/bdb/marketing-documents/cd_marker_handbook.pdf). To explore the utility of using natural ligands rather than mAbs to characterize receptors on immune cells, we produced CXCL chemokine-Fc fusions and characterized their interactions with receptors on transfected cells (Fig. 3*E*) and on immune cells (Fig. 4). As shown in Figure 3*E*, following transient transfection, COS cells expressing N-terminally FLAG-tagged CXCR2, a receptor for CXCL1, CXCL2, and several other CXCL chemokines, bind both CXCL2-Fc and anti-FLAG mAb (32, 33, 34, 35).

To test the utility of CXCL-Fc fusions for immunophenotyping, we used flow cytometry to analyze immune cells harvested from the spleens of 4-week-old control mice or mice that had received an intraperitoneal injection of 0.2 mg of lipopolysaccharide (LPS) 24 h prior to the analysis (Fig. 4). Inflammatory stimuli are known to increase the levels of some chemokine receptors (32). Primary binding reagents consisted of CXCL1-Fc, CXCL2-Fc, and a control Fc with no fusion protein partner, all produced in SFCM using the rabbit Fc expression plasmid. Mouse CXCL1 and CXCL2 share 71% amino acid identity, and both bind to CXCR2 (36, 37). Binding was detected with a donkey anti-rabbit phycoerythrin secondary antibody.

Colabeling of CXCL1-Fc or CXCL2-Fc with anti-CD19 (a marker for the B-cell lineage) shows that (1) nearly all CD19+ cells are labeled by CXCL1-Fc and CXCL2-Fc after LPS treatment, (2) the majority of cells that are labeled with CXCL1-Fc and CXCL2-Fc after LPS treatment are CD19+, (3) CD19+ cells exhibit moderate labeling by CXCL1-Fc in the absence of LPS treatment and ∼10-fold more intense labeling following LPS treatment (*blue arrows* in the third pair of panels), and (4) CD19+ cells exhibit minimal labeling by CXCL2-Fc in the absence of LPS treatment and >10-fold more intense labeling following LPS treatment (*blue arrows* in the fourth pair of panels). Colabeling with anti-CD11b (a marker for monocytes, macrophages, granulocytes, and NK cells) shows that (1) while most CD11b+ cells are labeled with CXCL1-Fc at moderate intensity, the intensity of labeling is minimally affected by LPS treatment and (2) approximately half of the CD11b+ cells are moderately labeled with CXCL2-Fc in the absence of LPS treatment, and that fraction increases following LPS treatment. The *green arrows* in the sixth and seventh pairs of panels show the increase in CXCL-Fc labeling of CD11b-negative cells following LPS treatment. The apparent reduction in the number of CD11b+ cells that do not bind CXCL2-Fc following LPS treatment could represent egress of these cells from the spleen.

A comparison of CXCL1-Fc and CXCL2-Fc binding in the absence of LPS treatment (*top row of panels* in Fig. 4) shows that CXCL1-Fc exhibits ∼10-fold greater binding to CD19+/CD11b- cells compared with CXCL2-Fc, providing indirect evidence for the presence on these cells of a receptor that binds preferentially to CXCL1. Presumably, this receptor is not CXCR2, which binds to both these ligands with similar affinities (37). Taken together, these data show that immunostaining with chemokine-Fc fusions in combination with flow cytometry can provide useful phenotyping information for immune cells and can reveal changes in the number of receptor sites on the cell surface.

## Discussion

The experiments described here demonstrate the production and use of a variety of fusion proteins in which diverse binding domain scaffolds are linked to Ig heavy chain Fc regions from chicken, goat, guinea pig, human, mouse, or rabbit. These reagents are easily produced on a laboratory scale by transient transfection of HEK293T cells. Their utility was demonstrated in immunostaining of tissue sections and transfected cells and in flow cytometry. Although Fc fusion proteins have been used extensively in research and as therapeutics (38, 39, 40, 41), to date, there has been no publicly available set of vectors that permit the convenient transfer of binding cassettes to Fc regions from multiple species. The six anti-GFP-DARPin-Fc expression plasmids described here fill this gap and have been deposited at Addgene (ID numbers: 231044-231049). They can be used as the starting point for other constructs by replacing the anti-GFP-DARPin segment with an alternate binding domain.

For immunostaining and flow cytometry applications, the commercial availability of large numbers of high-quality secondary antibody reagents has enabled researchers to focus their efforts on identifying and characterizing primary antibodies. While the number of commercially available primary antibodies is now estimated at more than six million (42), this abundance has been accompanied by questions related to quality and rigor of characterization (43, 44, 45, 46). Another challenge has been the high cost of many primary antibodies, a challenge that is magnified by the not infrequent realization that an antibody fails to perform as expected. One partial solution to these challenges is to make the greatest possible use of those antibodies (and non–antibody-binding proteins) that have been rigorously validated. The collection of plasmids developed here is a step in that direction, as it permits the construction of fusion proteins with the Fc domain from multiple species. This will enable validated binding proteins to be used in multiplexed experiments that would not be possible if these binding proteins were only available as a fusion to the Fc of a single species.

## Experimental procedures

### Mouse lines and LPS treatment

The following mouse lines were used: *VECad-CreER* (47) and *R26-LSL-SUN1-sfGFP* (14). For LPS treatment, mice (∼20 g body weight) received an intraperitoneal injection of 100 μl of 2 mg/ml LPS O111:B4 (Sigma; L2630) in PBS, 24 h prior to sacrifice. All mice were housed and handled according to the approved Institutional Animal Care and Use Committee protocol of the Johns Hopkins Medical Institutions (protocol no.: M022M375).

### Construction of Fc expression plasmids

The human IgG Fc gene segment was a gift of Dr Brian Seed (38). Chicken, goat, guinea pig, mouse, and rabbit Fc exons were PCR amplified from their corresponding genomic DNAs, with primer design based on complete or partial DNA sequences in the literature and/or GenBank. Where genome sequences were incomplete and intron lengths uncertain (chicken, goat, and guinea pig), PCR products encompassing some or all the exons were joined together with a second round of PCR to eliminate the intron between them. The pRK5 vector in which all the Fc segments were inserted uses a cytomegalovirus enhancer/promoter and has an intron in the 5′ UTR, which is present in all the Fc expression plasmids. Because chicken Fc does not bind to protein-A or protein-G, two versions of the chicken Fc expression plasmid were generated, with or without a C-terminal 8xHis tag.

### Construction of Fc fusions

Anti-Homer1 NB HS69 sequences were PCR amplified from plasmid 134716 (Addgene; (18)). Anti-Gephryn DARPin 27G2 sequences were PCR amplified from plasmid 199616 (Addgene; (19)). Anti-Synaptotagmin-6 scFv N270/47 sequences were PCR amplified from plasmid 190544 (Addgene; (20)). Alpha-BTX sequences were amplified from plasmid 69542 (Addgene). Anti-GFP DARPin 3G86.32 (12), PcTx1, CXCL1, and CXCL2 sequences were synthesized with optimal mammalian codons (by Integrated DNA Technologies) and then transferred to the various Fc vectors.

### Fc fusion protein production

For production of Fc fusion proteins, expression plasmids were transiently transfected into HEK293T cells using polyethyleneimine (PEI). A PEI stock solution (1 mg/ml) was prepared with either 25 kDa branched PEI (Sigma; catalog no.: 408727) or 25 kD linear PEI (Polysciences; catalog no.: 23966-2) by first dissolving PEI in endotoxin-free dH_2_O that had been preheated to ∼80 °C. The PEI solution was allowed to slowly cool to room temperature and then neutralized to pH ∼7.0 with concentrated NaOH (this step is optional), filter sterilized through a 0.22 μm filter, and aliquoted and stored at −80 °C. For protein production, 4 × 10^6^ HEK293T cells were seeded onto a gelatin-coated 10 cm dish in Dulbecco's modified Eagle's medium/F12 (1:1) medium with 10% fetal bovine serum and grown at 37 °C in a 5% CO_2_ atmosphere. The following day, when the cells were ∼60% confluent, the cells were changed to fresh medium for an additional 3 to 5 h incubation before transfection. In a sterile tube, 8 μg plasmid DNA was diluted in 1 ml serum-free medium (SFM), and then 24 μl 1 mg/ml PEI was added and the tube vortexed. After incubating for 15 min at room temperature, the DNA–PEI–SFM solution was added to the plate and dispersed with gentle rocking. After 1 day of incubation, the medium was removed, the cells were gently washed with three changes of sterile PBS, and 10 ml of SFM (Dulbecco's modified Eagle's medium/F12 [1:1]) was added. After an additional 2 days of incubation, the SFCM was collected, centrifuged to remove any detached cells or cell debris, and stored in aliquots at −80 °C.

Yields of the mammalian fusion proteins were estimated by capturing 0.5 ml of SFCM on protein-G magnetic beads, releasing the captured protein in SDS sample buffer at 95 °C, resolving the released protein by SDS-PAGE, and comparing the Coomassie Blue staining intensity with a dilution series of bovine serum albumin (BSA). With various fusion partners, yields of Fc fusions ranged from <0.01 μg/ml in SFCM (*i.e.*, undetectable by Coomassie Blue staining) to ∼2 μg/ml. Fc proteins without an N-terminal fusion partner were produced in the same manner using the Fc vector with an in-frame signal peptide but lacking an insert. 8xHis-tagged chicken Fc fusion proteins, which cannot be captured on protein A or protein G beads, were purified with Ni Sepharose Excel resin (Cytiva). Ten milliliter of SFCM was incubated with 100 μl prewashed resin (with a binding capacity >10 mg His-tagged protein/ml). After 2 h of gentle rotation at 4 °C, the resin was washed twice with wash buffer (20 mM sodium phosphate, 0.5 M NaCl, 20 mM imidazole, pH 7.4) to remove the unbound proteins and then eluted with 1.5 ml elution buffer (20 mM sodium phosphate, 0.5 M NaCl, 500 mM imidazole, pH 7.4) for 2 h with gentle rotation at 4 °C. The eluted chicken Fc protein was concentrated using a 10 kDa Amicon Ultra-4 centrifugal filter (MilliporeSigma). After centrifugation at 4000*g* for 20 min at 4 °C, 3 ml of storage buffer (10 mM Hepes, 150 mM NaCl, pH 7.4) was adding to the concentrated protein solution, and the concentration process repeated three times to reduce the concentration of residual imidazole. Aliquots were stored at −80 °C.

### Immunostaining of transfected COS and 293T cells

COS cells were cultured on gelatin-coated glass coverslips in the wells of a 24-well plate and transfected with the CXCR2-Tango plasmid (N-terminal FLAG-tagged CXCR2; 34; Addgene #66260) using PEI. For live cell staining, the medium was replaced 24 h after transfection with prechilled PBSC (PBS with 0.1 mM CaCl_2_) and then incubated at 4 °C for 2 h with CXCL2-Fc (rabbit or guinea pig) in SFCM diluted 1:50 in SFM with 1% BSA and anti-FLAG mouse mAb (catalog no.: MA1-91878; ThermoFisher). After 2 h, the coverslips were washed three times with 0.8 ml PBSC, for 10 min each at 4 °C. After washing, the coverslips were fixed with 1% PFA at room temperature for 1 h and then washed three times with PBSC to remove excess PFA. The coverslips were incubated overnight with Alexa488 goat anti-rabbit or goat anti–guinea pig secondary antibodies and Alexa594 goat antimouse secondary antibodies (ThermoFisher; diluted 1:500 in PBS containing 0.25% Triton X-100 (PBST) and 0.1 mM CaCl_2_ with 7% normal goat serum [NGS]) and with 4′,6-diamidino-2-phenylindole at 4 °C overnight. The following day, the coverslips were washed three times with PBSC and inverted onto glass slides with a drop of Flouromount-G (Southern Biotech; catalog no.: 0100-01). Slides were imaged using a Zeiss LSM700 confocal microscope with Zen software.

293T cells were cultured on gelatin-coated glass coverslips in the wells of a 24-well plate and transfected with a mammalian expression plasmid containing a nuclear-localized GFP (pMAT11) using PEI. About 24 h after transfection, the coverslips were washed carefully with PBS and fixed with 1% PFA for 30 min at room temperature. The fixed cells were washed with PBS three times to remove excess PFA, permeabilized with PBST for 15 min at room temperature and blocked with PBST + 1% BSA (PBSTB) for 30 min at room temperature. The coverslips were then incubated with anti-GFP-DARPin in SFCM that was diluted 1:50 in PBSTB overnight at 4 °C. The next day the coverslips were washed with PBS three times, incubated with the appropriate Alexa594-conjugated secondary antibodies (ThermoFisher; diluted 1:500 in PBSTB) and with 4′,6-diamidino-2-phenylindole at 4 °C overnight. The following day, the coverslips were washed three times with PBSC and inverted onto glass slides with a drop of Flouromount-G. Slides were imaged using a Zeiss LSM700 confocal microscope with Zen software.

### Tissue processing and immunohistochemistry

For retina sections, mouse eyes isolated immediately post-mortem were imbedded in Optimal Cutting Temperature compound (Tissue-Tek 4853) in plastic molds and frozen in dry ice. Embedded eyes were cut into 14 μm sections, and the glass slides were stored at −80 °C. For immunostaining, slides were first prewarmed to room temperature, fixed in 1% PFA at room temperature for 30 min, and washed in PBS to remove PFA before immunostaining.

For spine sections from P1 mice, the spine and proximal regions of the rib cage were dissected and washed with PBS and fixed in 2% PFA at 4 °C overnight. After washing in PBS for 2 to 4 h, the tissues were embedded in 3% agarose and sectioned in the transverse plane with a vibratome at a thickness of 200 μm.

For immunostaining, tissue sections (on glass slides for frozen sections or immersed in buffer for vibratome sections) were permeabilized in PBSTC (1× PBS, 1% Triton X-100, and 0.1 mM CaCl_2_) overnight at 4 °C. Sections were incubated for several hours in PBSTC containing 7% NGS (for non-goat Fc fusion proteins) and then incubated overnight at 4 °C with SFCM containing the Fc fusion protein(s) diluted 1:50 in PBSTC containing 7% NGS. Tissue sections were washed three times 2 h with PBSTC and then incubated overnight at 4 °C with the corresponding Alexa Fluor–conjugated secondary antibodies (ThermoFisher) diluted in 1× PBSTC containing 7% NGS (for non-goat Fc fusion proteins). The following day, tissue sections were washed three times with PBSTC, with each wash lasting 2 h. The washed sections were cover-slipped in Flouromount-G and imaged using a Zeiss LSM700 confocal microscope with Zen software.

### Flow cytometry with mouse splenocytes

Spleens from 4-week-old mice were collected and cut into small pieces. The tissues were gently homogenized with an AA size (4 ml) glass tissue homogenizer in prechilled PBSB (PBS containing 1% BSA) and filtered through a 40 μm cell strainer on ice. Cells were collected by centrifugation at 500*g* for 5 min at 4 °C. After discarding the supernatant, the pellet was resuspended in 1 ml ACK Lysis Buffer (Gibco; catalog no.: A1049201) to lyse erythrocytes. After a 2-min incubation at room temperature, the volume was brought up to 15 ml by addition of PBSB. The cells were collected by centrifugation at 500*g* for 5 min at 4 °C, and the pellet was resuspended in 2 ml PBSB. The cells were counted and diluted to a concentration of 10^7^ cells/ml using blocking buffer (PBSB containing 1% mouse serum). About 100 μl of the cell suspension (10^6^ cells) was added to each tube, and the incubation with blocking buffer was continued on ice with gentle vortexing every 10 min for a total of 30 min. Following this blocking step, 10 μl SFCM with the appropriate CXCL-Fc (rabbit) fusion protein was added to each tube for a 30-min incubation on ice, with gentle vortexing every 10 min. After 30 min, the cells were collected by centrifugation and resuspended in 1 ml PBSB buffer. This process was repeated three times to remove unbound Fc fusion protein. After washing, the cells were resuspended in 100 μl blocking buffer containing 1:200 diluted phycoerythrin-conjugated donkey F(ab’)_2_ anti-rabbit secondary antibody (emission maximum 576 nm; ThermoFisher; catalog no.: 12-4739-81) and either of two allophycocyanin eFluor 780–conjugated anti-CD marker antibodies (emission maximum 780 nm; ThermoFisher; anti-CD19, catalog no.: 47-0193-80; anti-CD11b, catalog no.: 47-0112-80). After a 30-min incubation with gentle vortexing every 10 min, the cells were washed three times with PBSB to remove unbound fluorescent secondary antibodies. The cells were analyzed using a high-speed flow cytometer (MoFlo XDP; Beckman Coulter). All analyses were gated for live cells and singlets.

## Data availability

All sequence data pertaining to the Fc constructs can be found in Figs. S1–S6.

## Supporting information

This article contains supporting information.

## Conflict of interest

The authors declare that they have no conflicts of interest with the contents of this article.
