# Supplementary material for: Utility of binding protein fusions to immunoglobulin heavy chain constant regions from mammalian and avian species
Source: J Biol Chem. 2025 Feb 18;301(4):108324. doi: 10.1016/j.jbc.2025.108324 (PMC11964738; doi:10.1016/j.jbc.2025.108324)
Supplement: Figures S1–S6 Legends [file mmc1.doc]

**Supplemental Figure Legends**

Figure S1. DNA and protein sequences for the coding region of the anti-GFP DARPin goat Fc fusion protein. The amino acid sequence of the DARPin is shown in green and the amino acid sequence of the hinge and Fc regions are shown in red. Useful restriction sites are shown in magenta.

Figure S2. DNA and protein sequences for the coding region of the anti-GFP DARPin guinea pig Fc fusion protein. The amino acid sequence of the DARPin is shown in green and the amino acid sequence of the hinge and Fc regions are shown in red. Useful restriction sites are shown in magenta.

Figure S3. DNA and protein sequences for the coding region of the anti-GFP DARPin human Fc fusion protein. The amino acid sequence of the DARPin is shown in green and the amino acid sequence of the hinge and Fc regions are shown in red. Useful restriction sites are shown in magenta.

Figure S4. DNA and protein sequences for the coding region of the anti-GFP DARPin mouse Fc fusion protein. The amino acid sequence of the DARPin is shown in green and the amino acid sequence of the hinge and Fc regions are shown in red. Useful restriction sites are shown in magenta.

Figure S5. DNA and protein sequences for the coding region of the anti-GFP DARPin rabbit Fc fusion protein. The amino acid sequence of the DARPin is shown in green and the amino acid sequence of the hinge and Fc regions are shown in red. Useful restriction sites are shown in magenta.

Figure S6. DNA and protein sequences for the coding region of the anti-GFP DARPin chicken Fc fusion protein. The amino acid sequence of the DARPin is shown in green and the amino acid sequence of the hinge, Fc, and His-tag regions are shown in red. Useful restriction sites are shown in magenta.
