# Supplementary material for: Utility of binding protein fusions to immunoglobulin heavy chain constant regions from mammalian and avian species
Source: J Biol Chem. 2025 Feb 18;301(4):108324. doi: 10.1016/j.jbc.2025.108324 (PMC11964738; doi:10.1016/j.jbc.2025.108324)
Supplement: Figure S1 [file mmc2.pdf]

Sequence: anti-GFP DARPin in Goat Fc – Figure S1

```
         10         20         30         40         50
GCGGCCGCCT GCACCTCGGT TCTATCGATT GAATTCCACC ATGGAGTGGG
CGCCGGCGGA CGTGGAGCCA AGATAGCTAA CTTAAGGTGG TACCTCACCC
                                             M  E  W

         60         70         80         90        100
GTTACCTGTT GGAAGTGACC TCGCTCCTAG CCGCCTTGGC GGTGCTACAG
CAATGGACAA CCTTCACTGG AGCGAGGATC GGCGGAACCG CCACGATGTC
G  Y  L  L   E  V  T   S  L  L   A  A  L  A   V  L  Q

        110        120        130        140        150
CGCTCTAGCG GCGCTGCCGC GGCTTCGGCC AAGGAGACGC GTGGTGTCGA
GCGAGATCGC CGCGACGGCG CCGAAGCCGG TTCCTCTGCG CACCACAGCT
 R  S  S   G  A  A  A   A  S  A   K  E  T   R  G  V  D

        160        170        180        190        200
CGGTGGTGAC CTGGGTAAGA AGCTGCTGGA AGCTGCTCGT GCTGGTCAGG
GCCACCACTG GACCCATTCT TCGACGACCT TCGACGAGCA CGACCAGTCC
  G  G  D   L  G  K   K  L  L  E   A  A  R   A  G  Q

        210        220        230        240        250
ACGACGAAGT TCGTATCCTG ATGGCTAACG GTGCCGATGT TAACGCACTT
TGCTGCTTCA AGCATAGGAC TACCGATTGC CACGGCTACA ATTGCGTGAA
D  D  E  V   R  I  L   M  A  N   G  A  D  V   N  A  L

        260        270        280        290        300
GACCGTTTTG GTCTTACTCC GCTGCACCTT GCTGCTCAGC GTGGCCACTT
CTGGCAAAAC CAGAATGAGG CGACGTGGAA CGACGAGTCG CACCGGTGAA
 D  R  F   G  L  T  P   L  H  L   A  A  Q   R  G  H  L

        310        320        330        340        350
AGAAATTGTT GAGGTTCTAC TGAAATGTGG TGCAGATGTA AATGCTGCTG
TCTTTAACAA CTCCAAGATG ACTTTACACC ACGTCTACAT TTACGACGAC
  E  I  V   E  V  L   L  K  C  G   A  D  V   N  A  A

        360        370        380        390        400
ACCTTTGGGG TCAGACTCCG CTGCACCTGG CTGCTACTGC TGGTCACTTA
TGGAAACCCC AGTCTGAGGC GACGTGGACC GACGATGACG ACCAGTGAAT
D  L  W  G   Q  T  P   L  H  L   A  A  T  A   G  H  L

        410        420        430        440        450
GAGATCGTCG AAGTCCTGCT GAAGTACGGT GCCGACGTGA ACGCACTCGA
CTCTAGCAGC TTCAGGACGA CTTCATGCCA CGGCTGCACT TGCGTGAGCT
 E  I  V   E  V  L  L   K  Y  G   A  D  V   N  A  L  D

        460        470        480        490        500
CCTTATTGGT AAGACTCCAC TGCACCTGAC TGCTATTGAT GGCCATCTGG
GGAATAACCA TTCTGAGGTG ACGTGGACTG ACGATAACTA CCGGTAGACC
  L  I  G   K  T  P   L  H  L  T   A  I  D   G  H  L

        510        520        530        540        550
AGATCGTCGA AGTCCTGCTA AAGCACGGTG CGGACGTCAA TGCTCAGGAC
TCTAGCAGCT TCAGGACGAT TTCGTGCCAC GCCTGCAGTT ACGAGTCCTG
E  I  V  E   V  L  L   K  H  G   A  D  V  N   A  Q  D
```

```
        560        570        580        590        600
AAATTCGGTA AGACCGCTTT CGACATCTCC ATCGACAATG GTAACGAGGA
TTTAAGCCAT TCTGGCGAAA GCTGTAGAGG TAGCTGTTAC CATTGCTCCT
 K  F  G   K  T  A  F   D  I  S   I  D  N   G  N  E  D

        610        620        630        640        650
CCTGGCTGAA ATCCTGCAAA AGCTTAATGG CGCGCCTGGT TCTGGTGGTT
GGACCGACTT TAGGACGTTT TCGAATTACC GCGCGGACCA AGACCACCAA
  L  A  E   I  L  Q   K  L  N  G   A  P  G   S  G  G


        660        670        680        690        700
CTGGTGACAA AACTCACACA TGCCCACCGT GCCCAGGTTC TGTCTTCATC
GACCACTGTT TTGAGTGTGT ACGGGTGGCA CGGGTCCAAG ACAGAAGTAG
S  G  D  K   T  H  T   C  P  P   C  P  G  S   V  F  I

        710        720        730        740        750
TTCCCCCCGA AACCCAAGGA CAGCCTCATG ATCACAGGAA CGCCCGAGGT
AAGGGGGGCT TTGGGTTCCT GTCGGAGTAC TAGTGTCCTT GCGGGCTCCA
 F  P  P   K  P  K  D   S  L  M   I  T  G   T  P  E  V

        760        770        780        790        800
CACGTGTGTG GTGGTGGACG TGGGCCAGGA CGACCCCGAG GTGCAGTTCT
GTGCACACAC CACCACCTGC ACCCGGTCCT GCTGGGGCTC CACGTCAAGA
  T  C  V   V  V  D   V  G  Q  D   D  P  E   V  Q  F

        810        820        830        840        850
CCTGGTTCGT GGACAACGTG GAGGTGCACA CGGCCAGGAC AAAGCCGAGA
GGACCAAGCA CCTGTTGCAC CTCCACGTGT GCCGGTCCTG TTTCGGCTCT
S  W  F  V   D  N  V   E  V  H   T  A  R  T   K  P  R

        860        870        880        890        900
GAGGAGCAGT TCAACAGCAC CTTCCGCGTG GTCAGCGCCC TGCCCATCCA
CTCCTCGTCA AGTTGTCGTG GAAGGCGCAC CAGTCGCGGG ACGGGTAGGT
 E  E  Q   F  N  S  T   F  R  V   V  S  A   L  P  I  Q

        910        920        930        940        950
GCACGACCAC TGGACTGGAG GGAAGGAGTT CAAGTGCAAG GTCAACAACA
CGTGCTGGTG ACCTGACCTC CCTTCCTCAA GTTCACGTTC CAGTTGTTGT
  H  D  H   W  T  G   G  K  E  F   K  C  K   V  N  N

        960        970        980        990       1000
AAGCCCTCCC GGCCCCCATC GTGAGGACCA TCTCCAGGGA CAAAGGTGGG
TTCGGGAGGG CCGGGGGTAG CACTCCTGGT AGAGGTCCCT GTTTCCACCC
K  A  L  P   A  P  I   V  R  T   I  S  R  D   K  G

       1010       1020       1030       1040       1050
CCAGGTGGGC GGGCCCAGGA AGGTCCCGTG GGCCAATCAG AGTGATTTCT
GGTCCACCCG CCCGGGTCCT TCCAGGGCAC CCGGTTAGTC TCACTAAAGA

       1060       1070       1080       1090       1100
GTGCTAACAG GCTTGCCTGT CCCCACAGGG CAGGCCCGGG AGCCGCAGGT
CACGATTGTC CGAACGGACA GGGGTGTCCC GTCCGGGCCC TCGGCGTCCA
                                     A  R   E  P  Q  V

       1110       1120       1130       1140       1150
GTACGTCCTG GCCCCACCCC AGGAAGAGCT CAGCAAAAGC ACGCTCAGCG
```

```
CATGCAGGAC CGGGGTGGGG TCCTTCTCGA GTCGTTTTCG TGCGAGTCGC
  Y  V  L   A  P  P   Q  E  E  L   S  K  S   T  L  S

      1160       1170       1180       1190       1200
TCACCTGCCT CGTCACCGGC TTCTACCCAG ACTACATCGC CGTGGAGTGG
AGTGGACGGA GCAGTGGCCG AAGATGGGTC TGATGTAGCG GCACCTCACC
V  T  C  L   V  T  G   F  Y  P   D  Y  I  A   V  E  W

      1210       1220       1230       1240       1250
CAGAGAGCGC GGCAGCCCGA GTCGGAGGAC AAGTACCGCA CGACCACATC
GTCTCTCGCG CCGTCGGGCT CAGCCTCCTG TTCATGGCGT GCTGGTGTAG
 Q  R  A   R  Q  P  E   S  E  D   K  Y  R   T  T  T  S

      1260       1270       1280       1290       1300
CCAGCTGGAC GCCGACGGCT CCTACTTCCT GTACAGCAGG CTCAGGGTGG
GGTCGACCTG CGGCTGCCGA GGATGAAGGA CATGTCGTCC GAGTCCCACC
  Q  L  D   A  D  G   S  Y  F  L   Y  S  R   L  R  V

      1310       1320       1330       1340       1350
ACAAGAGCAG CTGGCAGGAA GGAGACACCT ACGCGTGTGT GGTGATGCAC
TGTTCTCGTC GACCGTCCTT CCTCTGTGGA TGCGCACACA CCACTACGTG
D  K  S  S   W  Q  E   G  D  T   Y  A  C  V   V  M  H

      1360       1370       1380       1390       1400
GAGGCTCTGC ACAATCACTA CACACAGAAG TCCATCTCTA AGCCTCCGGG
CTCCGAGACG TGTTAGTGAT GTGTGTCTTC AGGTAGAGAT TCGGAGGCCC
 E  A  L   H  N  H  Y   T  Q  K   S  I  S   K  P  P  G

      1410
TAAATGAGGG CCCGAGCTT
ATTTACTCCC GGGCTCGAA
  K  *  G   P  E  L
```
