# Supplementary material for: Utility of binding protein fusions to immunoglobulin heavy chain constant regions from mammalian and avian species
Source: J Biol Chem. 2025 Feb 18;301(4):108324. doi: 10.1016/j.jbc.2025.108324 (PMC11964738; doi:10.1016/j.jbc.2025.108324)
Supplement: Figure S2 [file mmc3.pdf]

Sequence: anti-GFP DARPin in Guinea Pig Fc – Figure S2

```
          10         20         30         40         50
  GCGGCCGCCT GCACCTCGGT TCTATCGATT GAATTCCACC ATGGAGTGGG
  CGCCGGCGGA CGTGGAGCCA AGATAGCTAA CTTAAGGTGG TACCTCACCC
                                               M  E  W>

          60         70         80         90        100
  GTTACCTGTT GGAAGTGACC TCGCTCCTAG CCGCCTTGGC GGTGCTACAG
  CAATGGACAA CCTTCACTGG AGCGAGGATC GGCGGAACCG CCACGATGTC
  G  Y  L  L   E  V  T   S  L  L   A  A  L  A   V  L  Q

         110        120        130        140        150
  CGCTCTAGCG GCGCTGCCGC GGCTTCGGCC AAGGAGACGC GTGGTGTCGA
  GCGAGATCGC CGCGACGGCG CCGAAGCCGG TTCCTCTGCG CACCACAGCT
   R  S  S   G  A  A  A   A  S  A   K  E  T   R  G  V  D

         160        170        180        190        200
  CGGTGGTGAC CTGGGTAAGA AGCTGCTGGA AGCTGCTCGT GCTGGTCAGG
  GCCACCACTG GACCCATTCT TCGACGACCT TCGACGAGCA CGACCAGTCC
    G  G  D   L  G  K   K  L  L  E   A  A  R   A  G  Q

         210        220        230        240        250
  ACGACGAAGT TCGTATCCTG ATGGCTAACG GTGCCGATGT TAACGCACTT
  TGCTGCTTCA AGCATAGGAC TACCGATTGC CACGGCTACA ATTGCGTGAA
  D  D  E  V   R  I  L   M  A  N   G  A  D  V   N  A  L

         260        270        280        290        300
  GACCGTTTTG GTCTTACTCC GCTGCACCTT GCTGCTCAGC GTGGCCACTT
  CTGGCAAAAC CAGAATGAGG CGACGTGGAA CGACGAGTCG CACCGGTGAA
   D  R  F   G  L  T  P   L  H  L   A  A  Q   R  G  H  L

         310        320        330        340        350
  AGAAATTGTT GAGGTTCTAC TGAAATGTGG TGCAGATGTA AATGCTGCTG
  TCTTTAACAA CTCCAAGATG ACTTTACACC ACGTCTACAT TTACGACGAC
    E  I  V   E  V  L   L  K  C  G   A  D  V   N  A  A

         360        370        380        390        400
  ACCTTTGGGG TCAGACTCCG CTGCACCTGG CTGCTACTGC TGGTCACTTA
  TGGAAACCCC AGTCTGAGGC GACGTGGACC GACGATGACG ACCAGTGAAT
  D  L  W  G   Q  T  P   L  H  L   A  A  T  A   G  H  L

         410        420        430        440        450
  GAGATCGTCG AAGTCCTGCT GAAGTACGGT GCCGACGTGA ACGCACTCGA
  CTCTAGCAGC TTCAGGACGA CTTCATGCCA CGGCTGCACT TGCGTGAGCT
   E  I  V   E  V  L  L   K  Y  G   A  D  V   N  A  L  D

         460        470        480        490        500
  CCTTATTGGT AAGACTCCAC TGCACCTGAC TGCTATTGAT GGCCATCTGG
  GGAATAACCA TTCTGAGGTG ACGTGGACTG ACGATAACTA CCGGTAGACC
    L  I  G   K  T  P   L  H  L  T   A  I  D   G  H  L

         510        520        530        540        550
  AGATCGTCGA AGTCCTGCTA AAGCACGGTG CGGACGTCAA TGCTCAGGAC
  TCTAGCAGCT TCAGGACGAT TTCGTGCCAC GCCTGCAGTT ACGAGTCCTG
  E  I  V  E   V  L  L   K  H  G   A  D  V  N   A  Q  D
```

```
       560        570        580        590        600
AAATTCGGTA AGACCGCTTT CGACATCTCC ATCGACAATG GTAACGAGGA
TTTAAGCCAT TCTGGCGAAA GCTGTAGAGG TAGCTGTTAC CATTGCTCCT
 K  F  G   K  T  A  F   D  I  S   I  D  N   G  N  E  D

       610        620        630        640        650
CCTGGCTGAA ATCCTGCAAA AGCTTAATGG CGCGCCTGGT TCTGGTGGTT
GGACCGACTT TAGGACGTTT TCGAATTACC GCGCGGACCA AGACCACCAA
  L  A  E   I  L  Q   K  L  N  G   A  P  G   S  G  G

       660        670        680        690        700
CTGGTACATG TCCCAAGTGC CCACCTCCTG AAAACCTGGG TGGACCATCT
GACCATGTAC AGGGTTCACG GGTGGAGGAC TTTTGGACCC ACCTGGTAGA
S  G  T  C   P  K  C   P  P  P   E  N  L  G   G  P  S

       710        720        730        740        750
GTCTTCATCT TTCCCCCGAA GCCCAAGGAC ACGCTCATGA TCTCCCTGAC
CAGAAGTAGA AAGGGGGCTT CGGGTTCCTG TGCGAGTACT AGAGGGACTG
 V  F  I   F  P  P  K   P  K  D   T  L  M   I  S  L  T

       760        770        780        790        800
CCCTAGGGTC ACATGTGTGG TGGTAGATGT GAGCCAAGAT GAGCCTGAAG
GGGATCCCAG TGTACACACC ACCATCTACA CTCGGTTCTA CTCGGACTTC
  P  R  V   T  C  V   V  V  D  V   S  Q  D   E  P  E

       810        820        830        840        850
TCCAGTTCAC ATGGTTCGTG GACAACAAAC CGGTCGGCAA TGCTGAGACA
AGGTCAAGTG TACCAAGCAC CTGTTGTTTG GCCAGCCGTT ACGACTCTGT
V  Q  F  T   W  F  V   D  N  K   P  V  G  N   A  E  T

       860        870        880        890        900
AAGCCCCGAG TGGAGCAATA CAACACGACA TTCCGCGTGG AAAGTGTCCT
TTCGGGGCTC ACCTCGTTAT GTTGTGCTGT AAGGCGCACC TTTCACAGGA
 K  P  R   V  E  Q  Y   N  T  T   F  R  V   E  S  V  L

       910        920        930        940        950
CCCCATCCAG CACCAGGACT GGCTGAGGGG CAAGGAATTC AAGTGCAAGG
GGGGTAGGTC GTGGTCCTGA CCGACTCCCC GTTCCTTAAG TTCACGTTCC
  P  I  Q   H  Q  D   W  L  R  G   K  E  F   K  C  K

       960        970        980        990       1000
TCTACAACAA AGCCCTGCCA GCCCCCATAG AGAAGACCAT CTCCAAAACC
AGATGTTGTT TCGGGACGGT CGGGGGTATC TCTTCTGGTA GAGGTTTTGG
V  Y  N  K   A  L  P   A  P  I   E  K  T  I   S  K  T

      1010       1020       1030       1040       1050
AAAGGTGGGA GTGCAGTGCA GGCAGGTGGG AGATTGGGAG GCACTGTGGG
TTTCCACCCT CACGTCACGT CCGTCCACCC TCTAACCCTC CGTGACACCC
 K

      1060       1070       1080       1090       1100
GGTGAAATGG ACAGGCCCTC CATGGCCAGC CCTCCATCCC TGGCATTGAA
CCACTTTACC TGTCCGGGAG GTACCGGTCG GGAGGTAGGG ACCGTAACTT

      1110       1120       1130       1140       1150
CATGTGCTGA TCTCTGTCCC ACAGGGGCTC CCCGCATGCC AGATGTGTAC
```

```
GTACACGACT AGAGACAGGG TGTCCCCGAG GGGCGTACGG TCTACACATG
                        G   A   P   R   M   P   D   V   Y

       1160       1170       1180       1190       1200
ACCCTTCCCC CGTCCCGAGA CGAGCTATCC AAGAGCAAAG TCAGTGTGAC
TGGGAAGGGG GCAGGGCTCT GCTCGATAGG TTCTCGTTTC AGTCACACTG
 T  L  P   P  S  R  D   E  L  S   K  S  K   V  S  V  T

       1210       1220       1230       1240       1250
CTGCCTGATC ATCAACTTCT TTCCTGCCGA CATCCACGTG GAGTGGGCCA
GACGGACTAG TAGTTGAAGA AAGGACGGCT GTAGGTGCAC CTCACCCGGT
  C  L  I   I  N  F   F  P  A  D   I  H  V   E  W  A

       1260       1270       1280       1290       1300
GCAATAGGGT TCCAGTGAGT GAGAAGGAAT ACAAGAACAC CCCACCCATT
CGTTATCCCA AGGTCACTCA CTCTTCCTTA TGTTCTTGTG GGGTGGGTAA
S  N  R  V   P  V  S   E  K  E   Y  K  N  T   P  P  I

       1310       1320       1330       1340       1350
GAGGACGCTG ACGGGTCCTA CTTCCTCTAC AGCAAGCTCA CTGTGGATAA
CTCCTGCGAC TGCCCAGGAT GAAGGAGATG TCGTTCGAGT GACACCTATT
 E  D  A   D  G  S  Y   F  L  Y   S  K  L   T  V  D  K

       1360       1370       1380       1390       1400
GAGCGCGTGG GATCAGGGAA CCGTCTACAC CTGCTCCGTG ATGCATGAAG
CTCGCGCACC CTAGTCCCTT GGCAGATGTG GACGAGGCAC TACGTACTTC
  S  A  W   D  Q  G   T  V  Y  T   C  S  V   M  H  E

       1410       1420       1430       1440       1450
CCCTGCACAA TCATGTCACT CAGAAGGCCA TCTCCCGCTC TCCGGGTAAA
GGGACGTGTT AGTACAGTGA GTCTTCCGGT AGAGGGCGAG AGGCCCATTT
A  L  H  N   H  V  T   Q  K  A   I  S  R  S   P  G  K

       1460       1470       1480       1490       1500
TGAGGGCCCG AGCTTGGCCG CCATGGCCCA ACTTGTTTAT TGCAGCTTAA
ACTCCCGGGC TCGAACCGGC GGTACCGGGT TGAACAAATA ACGTCGAATT
 *
```
