# Supplementary material for: Utility of binding protein fusions to immunoglobulin heavy chain constant regions from mammalian and avian species
Source: J Biol Chem. 2025 Feb 18;301(4):108324. doi: 10.1016/j.jbc.2025.108324 (PMC11964738; doi:10.1016/j.jbc.2025.108324)
Supplement: Figure S3 [file mmc4.pdf]

Sequence: anti-GFP DARPin in Human Fc - Figure S3

```
          10         20         30         40         50
  GCGGCCGCCT GCACCTCGGT TCTATCGATT GAATTCCACC ATGGAGTGGG
  CGCCGGCGGA CGTGGAGCCA AGATAGCTAA CTTAAGGTGG TACCTCACCC
                                               M  E  W

          60         70         80         90        100
  GTTACCTGTT GGAAGTGACC TCGCTCCTAG CCGCCTTGGC GGTGCTACAG
  CAATGGACAA CCTTCACTGG AGCGAGGATC GGCGGAACCG CCACGATGTC
  G  Y  L  L   E  V  T   S  L  L   A  A  L  A   V  L  Q

         110        120        130        140        150
  CGCTCTAGCG GCGCTGCCGC GGCTTCGGCC AAGGAGACGC GTGGTGTCGA
  GCGAGATCGC CGCGACGGCG CCGAAGCCGG TTCCTCTGCG CACCACAGCT
   R  S  S   G  A  A  A   A  S  A   K  E  T   R  G  V  D

         160        170        180        190        200
  CGGTGGTGAC CTGGGTAAGA AGCTGCTGGA AGCTGCTCGT GCTGGTCAGG
  GCCACCACTG GACCCATTCT TCGACGACCT TCGACGAGCA CGACCAGTCC
    G  G  D   L  G  K   K  L  L  E   A  A  R   A  G  Q

         210        220        230        240        250
  ACGACGAAGT TCGTATCCTG ATGGCTAACG GTGCCGATGT TAACGCACTT
  TGCTGCTTCA AGCATAGGAC TACCGATTGC CACGGCTACA ATTGCGTGAA
  D  D  E  V   R  I  L   M  A  N   G  A  D  V   N  A  L

         260        270        280        290        300
  GACCGTTTTG GTCTTACTCC GCTGCACCTT GCTGCTCAGC GTGGCCACTT
  CTGGCAAAAC CAGAATGAGG CGACGTGGAA CGACGAGTCG CACCGGTGAA
   D  R  F   G  L  T  P   L  H  L   A  A  Q   R  G  H  L

         310        320        330        340        350
  AGAAATTGTT GAGGTTCTAC TGAAATGTGG TGCAGATGTA AATGCTGCTG
  TCTTTAACAA CTCCAAGATG ACTTTACACC ACGTCTACAT TTACGACGAC
    E  I  V   E  V  L   L  K  C  G   A  D  V   N  A  A

         360        370        380        390        400
  ACCTTTGGGG TCAGACTCCG CTGCACCTGG CTGCTACTGC TGGTCACTTA
  TGGAAACCCC AGTCTGAGGC GACGTGGACC GACGATGACG ACCAGTGAAT
  D  L  W  G   Q  T  P   L  H  L   A  A  T  A   G  H  L

         410        420        430        440        450
  GAGATCGTCG AAGTCCTGCT GAAGTACGGT GCCGACGTGA ACGCACTCGA
  CTCTAGCAGC TTCAGGACGA CTTCATGCCA CGGCTGCACT TGCGTGAGCT
   E  I  V   E  V  L  L   K  Y  G   A  D  V   N  A  L  D

         460        470        480        490        500
  CCTTATTGGT AAGACTCCAC TGCACCTGAC TGCTATTGAT GGCCATCTGG
  GGAATAACCA TTCTGAGGTG ACGTGGACTG ACGATAACTA CCGGTAGACC
    L  I  G   K  T  P   L  H  L  T   A  I  D   G  H  L

         510        520        530        540        550
  AGATCGTCGA AGTCCTGCTA AAGCACGGTG CGGACGTCAA TGCTCAGGAC
  TCTAGCAGCT TCAGGACGAT TTCGTGCCAC GCCTGCAGTT ACGAGTCCTG
  E  I  V  E   V  L  L   K  H  G   A  D  V  N   A  Q  D
```

```
       560        570        580        590        600
AAATTCGGTA AGACCGCTTT CGACATCTCC ATCGACAATG GTAACGAGGA
TTTAAGCCAT TCTGGCGAAA GCTGTAGAGG TAGCTGTTAC CATTGCTCCT
 K  F  G   K  T  A  F   D  I  S   I  D  N   G  N  E  D

       610        620        630        640        650
CCTGGCTGAA ATCCTGCAAA AGCTTAATGG CGCGCCTGGG CCCCGCACCG
GGACCGACTT TAGGACGTTT TCGAATTACC GCGCGGACCC GGGGCGTGGC
  L  A  E   I  L  Q   K  L  N  G   A  P  G   P  R  T>

       660        670        680        690        700
ACCTCACCAC GGCCGCGCCC AGCCCACCGC GCCGCCTGCC TCCGCCGCCT
TGGAGTGGTG CCGGCGCGGG TCGGGTGGCG CGGCGGACGG AGGCGGCGGA
D  L  T  T   A  A  P   S  P  P   R  R  L  P   P  P  P>

       710        720        730        740        750
CCTCCCAAGC TTGGAGGTGG AGGGGATCCC GAGGGTGAGT ACTAAGCTTC
GGAGGGTTCG AACCTCCACC TCCCCTAGGG CTCCCACTCA TGATTCGAAG
 P  P  K   L  G  G  G   G  D  P   E

       760        770        780        790        800
AGCGCTCCTG CCTGGACGCA TCCCGGCTAT GCAGCCCCAG TCCAGGGCAG
TCGCGAGGAC GGACCTGCGT AGGGCCGATA CGTCGGGGTC AGGTCCCGTC

       810        820        830        840        850
CAAGGCAGGC CCCGTCTGCC TCTTCACCCG GAGGCCTCTG CCCGCCCCAC
GTTCCGTCCG GGGCAGACGG AGAAGTGGGC CTCCGGAGAC GGGCGGGGTG

       860        870        880        890        900
TCATGCTCAG GGAGAGGGTC TTCTGGCTTT TTCCCCAGGC TCTGGGCAGG
AGTACGAGTC CCTCTCCCAG AAGACCGAAA AAGGGGTCCG AGACCCGTCC

       910        920        930        940        950
CACAGGCTAG GTGCCCCTAA CCCAGGCCCT GCACACAAAG GGGCAGGTGC
GTGTCCGATC CACGGGGATT GGGTCCGGGA CGTGTGTTTC CCCGTCCACG

       960        970        980        990       1000
TGGGCTCAGA CCTGCCAAGA GCCATATCCG GGAGGACCCT GCCCCTGACC
ACCCGAGTCT GGACGGTTCT CGGTATAGGC CCTCCTGGGA CGGGGACTGG

      1010       1020       1030       1040       1050
TAAGCCCACC CCAAAGGCCA AACTCTCCAC TCCCTCAGCT CGGACACCTT
ATTCGGGTGG GGTTTCCGGT TTGAGAGGTG AGGGAGTCGA GCCTGTGGAA

      1060       1070       1080       1090       1100
CTCTCCTCCC AGATTCCAGT AACTCCCAAT CTTCTCTCTG CAGAGCCCAA
GAGAGGAGGG TCTAAGGTCA TTGAGGGTTA GAAGAGAGAC GTCTCGGGTT
                                               E  P  K

      1110       1120       1130       1140       1150
ATCTTGTGAC AAAACTCACA CATGCCCACC GTGCCCAGGT AAGCCAGCCC
TAGAACACTG TTTTGAGTGT GTACGGGTGG CACGGGTCCA TTCGGTCGGG
  S  C  D   K  T  H   T  C  P  P   C  P

      1160       1170       1180       1190       1200
AGGCCTCGCC CTCCAGCTCA AGGCGGGACA GGTGCCCTAG AGTAGCCTGC
TCCGGAGCGG GAGGTCGAGT TCCGCCCTGT CCACGGGATC TCATCGGACG
```

```
      1210       1220       1230       1240       1250
ATCCAGGGAC AGGCCCCAGC CGGGTGCTGA CACGTCCACC TCCATCTCTT
TAGGTCCCTG TCCGGGGTCG GCCCACGACT GTGCAGGTGG AGGTAGAGAA

      1260       1270       1280       1290       1300
CCTCAGCACC TGAACTCCTG GGGGGACCGT CAGTCTTCCT CTTCCCCCCA
GGAGTCGTGG ACTTGAGGAC CCCCCTGGCA GTCAGAAGGA GAAGGGGGGT
      A  P   E  L  L   G  G  P   S  V  F  L   F  P  P

      1310       1320       1330       1340       1350
AAACCCAAGG ACACCCTCAT GATCTCCCGG ACCCCTGAGG TCACATGCGT
TTTGGGTTCC TGTGGGAGTA CTAGAGGGCC TGGGGACTCC AGTGTACGCA
 K  P  K   D  T  L  M   I  S  R   T  P  E   V  T  C  V

      1360       1370       1380       1390       1400
GGTGGTGGAC GTGAGCCACG AAGACCCTGA GGTCAAGTTC AACTGGTACG
CCACCACCTG CACTCGGTGC TTCTGGGACT CCAGTTCAAG TTGACCATGC
  V  V  D   V  S  H   E  D  P  E   V  K  F   N  W  Y

      1410       1420       1430       1440       1450
TGGACGGCGT GGAGGTGCAT AATGCCAAGA CAAAGCCGCG GGAGGAGCAG
ACCTGCCGCA CCTCCACGTA TTACGGTTCT GTTTCGGCGC CCTCCTCGTC
V  D  G  V   E  V  H   N  A  K   T  K  P  R   E  E  Q

      1460       1470       1480       1490       1500
TACAACAGCA CGTACCGTGT GGTCAGCGTC CTCACCGTCC TGCACCAGGA
ATGTTGTCGT GCATGGCACA CCAGTCGCAG GAGTGGCAGG ACGTGGTCCT
 Y  N  S   T  Y  R  V   V  S  V   L  T  V   L  H  Q  D

      1510       1520       1530       1540       1550
CTGGCTGAAT GGCAAGGAGT ACAAGTGCAA GGTCTCCAAC AAAGCCCTCC
GACCGACTTA CCGTTCCTCA TGTTCACGTT CCAGAGGTTG TTTCGGGAGG
  W  L  N   G  K  E   Y  K  C  K   V  S  N   K  A  L

      1560       1570       1580       1590       1600
CAGCCCCCAT CGAGAAAACC ATCTCCAAAG CCAAAGGTGG GACCCGTGGG
GTCGGGGGTA GCTCTTTTGG TAGAGGTTTC GGTTTCCACC CTGGGCACCC
P  A  P  I   E  K  T   I  S  K   A  K

      1610       1620       1630       1640       1650
GTGCGAGGGC CACATGGACA GAGGCCGGCT CGGCCCACCC TCTGCCCTGA
CACGCTCCCG GTGTACCTGT CTCCGGCCGA GCCGGGTGGG AGACGGGACT

      1660       1670       1680       1690       1700
GAGTGACCGC TGTACCAACC TCTGTCCCTA CAGGGCAGCC CCGAGAACCA
CTCACTGGCG ACATGGTTGG AGACAGGGAT GTCCCGTCGG GGCTCTTGGT
                                    G  Q  P   R  E  P

      1710       1720       1730       1740       1750
CAGGTGTACA CCCTGCCCCC ATCCCGGGAT GAGCTGACCA AGAACCAGGT
GTCCACATGT GGGACGGGGG TAGGGCCCTA CTCGACTGGT TCTTGGTCCA
 Q  V  Y   T  L  P  P   S  R  D   E  L  T   K  N  Q  V

      1760       1770       1780       1790       1800
CAGCCTGACC TGCCTGGTCA AAGGCTTCTA TCCCAGCGAC ATCGCCGTGG
GTCGGACTGG ACGGACCAGT TTCCGAAGAT AGGGTCGCTG TAGCGGCACC
```

```
   S  L  T   C  L  V   K  G  F  Y   P  S  D   I  A  V

      1810       1820       1830       1840       1850
AGTGGGAGAG CAATGGGCAG CCGGAGAACA ACTACAAGAC CACGCCTCCC
TCACCCTCTC GTTACCCGTC GGCCTCTTGT TGATGTTCTG GTGCGGAGGG
E  W  E  S   N  G  Q   P  E  N   N  Y  K  T   T  P  P

      1860       1870       1880       1890       1900
GTGCTGGACT CCGACGGCTC CTTCTTCCTC TACAGCAAGC TCACCGTGGA
CACGACCTGA GGCTGCCGAG GAAGAAGGAG ATGTCGTTCG AGTGGCACCT
 V  L  D   S  D  G  S   F  F  L   Y  S  K   L  T  V  D

      1910       1920       1930       1940       1950
CAAGAGCAGG TGGCAGCAGG GGAACGTCTT CTCATGCTCC GTGATGCATG
GTTCTCGTCC ACCGTCGTCC CCTTGCAGAA GAGTACGAGG CACTACGTAC
  K  S  R   W  Q  Q   G  N  V  F   S  C  S   V  M  H

      1960       1970       1980       1990       2000
AGGCTCTGCA CAACCACTAC ACGCAGAAGA GCCTCTCCCT GTCTCCGGGT
TCCGAGACGT GTTGGTGATG TGCGTCTTCT CGGAGAGGGA CAGAGGCCCA
E  A  L  H   N  H  Y   T  Q  K   S  L  S  L   S  P  G

      2010       2020       2030       2040
AAATGAGTGC GACGGCCTTC GGGCATGCAG CTTGGCCGCC ATGGCCCAA
TTTACTCACG CTGCCGGAAG CCCGTACGTC GAACCGGCGG TACCGGGTT
 K  *
```
