# Supplementary material for: Utility of binding protein fusions to immunoglobulin heavy chain constant regions from mammalian and avian species
Source: J Biol Chem. 2025 Feb 18;301(4):108324. doi: 10.1016/j.jbc.2025.108324 (PMC11964738; doi:10.1016/j.jbc.2025.108324)
Supplement: Figure S4 [file mmc5.pdf]

Sequence: anti-GFP DARPin in Mouse Fc – Figure S4

```
         10         20         30         40         50
GCGGCCGCCT GCACCTCGGT TCTATCGATT GAATTCCACC ATGGAGTGGG
CGCCGGCGGA CGTGGAGCCA AGATAGCTAA CTTAAGGTGG TACCTCACCC
                                             M  E  W

         60         70         80         90        100
GTTACCTGTT GGAAGTGACC TCGCTCCTAG CCGCCTTGGC GGTGCTACAG
CAATGGACAA CCTTCACTGG AGCGAGGATC GGCGGAACCG CCACGATGTC
G  Y  L  L   E  V  T   S  L  L  A  A  L  A   V  L  Q

        110        120        130        140        150
CGCTCTAGCG GCGCTGCCGC GGCTTCGGCC AAGGAGACGC GTGGTGTCGA
GCGAGATCGC CGCGACGGCG CCGAAGCCGG TTCCTCTGCG CACCACAGCT
 R  S  S   G  A  A  A   A  S  A   K  E  T   R  G  V  D

        160        170        180        190        200
CGGTGGTGAC CTGGGTAAGA AGCTGCTGGA AGCTGCTCGT GCTGGTCAGG
GCCACCACTG GACCCATTCT TCGACGACCT TCGACGAGCA CGACCAGTCC
  G  G  D   L  G  K   K  L  L  E   A  A  R   A  G  Q

        210        220        230        240        250
ACGACGAAGT TCGTATCCTG ATGGCTAACG GTGCCGATGT TAACGCACTT
TGCTGCTTCA AGCATAGGAC TACCGATTGC CACGGCTACA ATTGCGTGAA
D  D  E  V   R  I  L   M  A  N   G  A  D  V   N  A  L

        260        270        280        290        300
GACCGTTTTG GTCTTACTCC GCTGCACCTT GCTGCTCAGC GTGGCCACTT
CTGGCAAAAC CAGAATGAGG CGACGTGGAA CGACGAGTCG CACCGGTGAA
 D  R  F   G  L  T  P   L  H  L   A  A  Q   R  G  H  L

        310        320        330        340        350
AGAAATTGTT GAGGTTCTAC TGAAATGTGG TGCAGATGTA AATGCTGCTG
TCTTTAACAA CTCCAAGATG ACTTTACACC ACGTCTACAT TTACGACGAC
  E  I  V   E  V  L   L  K  C  G   A  D  V   N  A  A

        360        370        380        390        400
ACCTTTGGGG TCAGACTCCG CTGCACCTGG CTGCTACTGC TGGTCACTTA
TGGAAACCCC AGTCTGAGGC GACGTGGACC GACGATGACG ACCAGTGAAT
D  L  W  G   Q  T  P   L  H  L   A  A  T  A   G  H  L

        410        420        430        440        450
GAGATCGTCG AAGTCCTGCT GAAGTACGGT GCCGACGTGA ACGCACTCGA
CTCTAGCAGC TTCAGGACGA CTTCATGCCA CGGCTGCACT TGCGTGAGCT
 E  I  V   E  V  L  L   K  Y  G   A  D  V   N  A  L  D

        460        470        480        490        500
CCTTATTGGT AAGACTCCAC TGCACCTGAC TGCTATTGAT GGCCATCTGG
GGAATAACCA TTCTGAGGTG ACGTGGACTG ACGATAACTA CCGGTAGACC
  L  I  G   K  T  P   L  H  L  T   A  I  D   G  H  L

        510        520        530        540        550
AGATCGTCGA AGTCCTGCTA AAGCACGGTG CGGACGTCAA TGCTCAGGAC
TCTAGCAGCT TCAGGACGAT TTCGTGCCAC GCCTGCAGTT ACGAGTCCTG
E  I  V  E   V  L  L   K  H  G   A  D  V  N   A  Q  D
```

```
       560        570        580        590        600
AAATTCGGTA AGACCGCTTT CGACATCTCC ATCGACAATG GTAACGAGGA
TTTAAGCCAT TCTGGCGAAA GCTGTAGAGG TAGCTGTTAC CATTGCTCCT
 K  F  G   K  T  A  F   D  I  S   I  D  N   G  N  E  D

       610        620        630        640        650
CCTGGCTGAA ATCCTGCAAA AGCTTAATGG CGCGCCTGGT TCTGGTGGTT
GGACCGACTT TAGGACGTTT TCGAATTACC GCGCGGACCA AGACCACCAA
  L  A  E   I  L  Q   K  L  N  G   A  P  G   S  G  G

       660        670        680        690        700
CTGGTGGTCC TGCTCCTCCA TGCAAATGCC CAGGTAAGTC ACTAGACCAG
GACCACCAGG ACGAGGAGGT ACGTTTACGG GTCCATTCAG TGATCTGGTC
S  G  G  P   A  P  P   C  K  C   P  G

       710        720        730        740        750
AGCTCCACTC CCGGGAGAAT GGTAAGTGCT GTAAACATCC CTGCACTAGA
TCGAGGTGAG GGCCCTCTTA CCATTCACGA CATTTGTAGG GACGTGATCT

       760        770        780        790        800
GGATAAGCCA TGTACAGATC CATTTCCATC TCTCCTCATC AGCACCTAAC
CCTATTCGGT ACATGTCTAG GTAAAGGTAG AGAGGAGTAG TCGTGGATTG
                                                P  N

       810        820        830        840        850
CTCTTGGGTG GACCATCCGT CTTCATCTTC CCTCCAAAGA TCAAGGATGT
GAGAACCCAC CTGGTAGGCA GAAGTAGAAG GGAGGTTTCT AGTTCCTACA
 L  L  G   G  P  S  V   F  I  F   P  P  K   I  K  D  V

       860        870        880        890        900
ACTCATGATC TCCCTGAGCC CCATAGTCAC ATGTGTGGTG GTGGATGTGA
TGAGTACTAG AGGGACTCGG GGTATCAGTG TACACACCAC CACCTACACT
  L  M  I   S  L  S   P  I  V  T   C  V  V   V  D  V

       910        920        930        940        950
GCGAGGATGA CCCAGATGTC CAGATCAGCT GGTTTGTGAA CAACGTGGAA
CGCTCCTACT GGGTCTACAG GTCTAGTCGA CCAAACACTT GTTGCACCTT
S  E  D  D   P  D  V   Q  I  S   W  F  V  N   N  V  E

       960        970        980        990       1000
GTACACACAG CTCAGACACA AACCCATAGA GAGGATTACA ACAGTACTCT
CATGTGTGTC GAGTCTGTGT TTGGGTATCT CTCCTAATGT TGTCATGAGA
 V  H  T   A  Q  T  Q   T  H  R   E  D  Y   N  S  T  L

      1010       1020       1030       1040       1050
CCGGGTGGTC AGTGCCCTCC CCATCCAGCA CCAGGACTGG ATGAGTGGCA
GGCCCACCAG TCACGGGAGG GGTAGGTCGT GGTCCTGACC TACTCACCGT
  R  V  V   S  A  L   P  I  Q  H   Q  D  W   M  S  G

      1060       1070       1080       1090       1100
AGGAGTTCAA ATGCAAGGTC AACAACAAAG ACCTCCCAGC GCCCATCGAG
TCCTCAAGTT TACGTTCCAG TTGTTGTTTC TGGAGGGTCG CGGGTAGCTC
K  E  F  K   C  K  V   N  N  K   D  L  P  A   P  I  E

      1110       1120       1130       1140       1150
AGAACCATCT CAAAACCCAA AGGTGAGAGC TGCAGCCTGA CTGCATGGGG
TCTTGGTAGA GTTTTGGGTT TCCACTCTCG ACGTCGGACT GACGTACCCC
```

```
 R  T  I   S  K  P  K

      1160      1170      1180      1190      1200
GCTGGGATGG GCATAAGGAT AAAGGTCTGT GTGGACAGCC TTCTGCTTCA
CGACCCTACC CGTATTCCTA TTTCCAGACA CACCTGTCGG AAGACGAAGT

      1210      1220      1230      1240      1250
GCCATGACCT TTGTGTATGT TTCTACCCTC ACAGGGTCAG TAAGAGCTCC
CGGTACTGGA AACACATACA AAGATGGGAG TGTCCCAGTC ATTCTCGAGG
                                     G  S   V  R  A  P

      1260      1270      1280      1290      1300
ACAGGTATAT GTCTTGCCTC CACCAGAAGA AGAGATGACT AAGAAACAGG
TGTCCATATA CAGAACGGAG GTGGTCTTCT TCTCTACTGA TTCTTTGTCC
  Q  V  Y   V  L  P   P  P  E  E   E  M  T   K  K  Q

      1310      1320      1330      1340      1350
TCACTCTGAC CTGCATGGTC ACAGACTTCA TGCCTGAAGA CATTTACGTG
AGTGAGACTG GACGTACCAG TGTCTGAAGT ACGGACTTCT GTAAATGCAC
V  T  L  T   C  M  V   T  D  F   M  P  E  D   I  Y  V

      1360      1370      1380      1390      1400
GAGTGGACCA ACAACGGGAA AACAGAGCTA AACTACAAGA ACACTGAACC
CTCACCTGGT TGTTGCCCTT TTGTCTCGAT TTGATGTTCT TGTGACTTGG
 E  W  T   N  N  G  K   T  E  L   N  Y  K   N  T  E  P

      1410      1420      1430      1440      1450
AGTCCTGGAC TCTGATGGTT CTTACTTCAT GTACAGCAAG CTGAGAGTGG
TCAGGACCTG AGACTACCAA GAATGAAGTA CATGTCGTTC GACTCTCACC
  V  L  D   S  D  G   S  Y  F  M   Y  S  K   L  R  V

      1460      1470      1480      1490      1500
AAAAGAAGAA CTGGGTGGAA AGAAATAGCT ACTCCTGTTC AGTGGTCCAC
TTTTCTTCTT GACCCACCTT TCTTTATCGA TGAGGACAAG TCACCAGGTG
E  K  K  N   W  V  E   R  N  S   Y  S  C  S   V  V  H

      1510      1520      1530      1540      1550
GAGGGTCTGC ACAATCACCA CACGACTAAG AGCTTCTCCC GGACTCCGGG
CTCCCAGACG TGTTAGTGGT GTGCTGATTC TCGAAGAGGG CCTGAGGCCC
 E  G  L   H  N  H  H   T  T  K   S  F  S   R  T  P  G

      1560      1570      1580
TAAATGAGCT CAGCACCCAC AAAACTCTCA GGGGCCCGA
ATTTACTCGA GTCGTGGGTG TTTTGAGAGT CCCCGGGCT
  K  *
```
