# Supplementary material for: Utility of binding protein fusions to immunoglobulin heavy chain constant regions from mammalian and avian species
Source: J Biol Chem. 2025 Feb 18;301(4):108324. doi: 10.1016/j.jbc.2025.108324 (PMC11964738; doi:10.1016/j.jbc.2025.108324)
Supplement: Figure S5 [file mmc6.pdf]

Sequence: anti-GFP DARPin in Rabbit Fc – Figure S5

```
          10         20         30         40         50
GCGGCCGCCT GCACCTCGGT TCTATCGATT GAATTCCACC ATGGAGTGGG
CGCCGGCGGA CGTGGAGCCA AGATAGCTAA CTTAAGGTGG TACCTCACCC
                                             M  E  W

          60         70         80         90        100
GTTACCTGTT GGAAGTGACC TCGCTCCTAG CCGCCTTGGC GGTGCTACAG
CAATGGACAA CCTTCACTGG AGCGAGGATC GGCGGAACCG CCACGATGTC
G  Y  L  L   E  V  T   S  L  L  A  A  L  A   V  L  Q

         110        120        130        140        150
CGCTCTAGCG GCGCTGCCGC GGCTTCGGCC AAGGAGACGC GTGGTGTCGA
GCGAGATCGC CGCGACGGCG CCGAAGCCGG TTCCTCTGCG CACCACAGCT
 R  S  S   G  A  A  A   A  S  A   K  E  T   R  G  V  D

         160        170        180        190        200
CGGTGGTGAC CTGGGTAAGA AGCTGCTGGA AGCTGCTCGT GCTGGTCAGG
GCCACCACTG GACCCATTCT TCGACGACCT TCGACGAGCA CGACCAGTCC
  G  G  D   L  G  K   K  L  L  E   A  A  R   A  G  Q

         210        220        230        240        250
ACGACGAAGT TCGTATCCTG ATGGCTAACG GTGCCGATGT TAACGCACTT
TGCTGCTTCA AGCATAGGAC TACCGATTGC CACGGCTACA ATTGCGTGAA
D  D  E  V   R  I  L   M  A  N  G  A  D  V   N  A  L

         260        270        280        290        300
GACCGTTTTG GTCTTACTCC GCTGCACCTT GCTGCTCAGC GTGGCCACTT
CTGGCAAAAC CAGAATGAGG CGACGTGGAA CGACGAGTCG CACCGGTGAA
 D  R  F   G  L  T  P   L  H  L   A  A  Q   R  G  H  L

         310        320        330        340        350
AGAAATTGTT GAGGTTCTAC TGAAATGTGG TGCAGATGTA AATGCTGCTG
TCTTTAACAA CTCCAAGATG ACTTTACACC ACGTCTACAT TTACGACGAC
  E  I  V   E  V  L   L  K  C  G   A  D  V   N  A  A

         360        370        380        390        400
ACCTTTGGGG TCAGACTCCG CTGCACCTGG CTGCTACTGC TGGTCACTTA
TGGAAACCCC AGTCTGAGGC GACGTGGACC GACGATGACG ACCAGTGAAT
D  L  W  G   Q  T  P   L  H  L  A  A  T  A   G  H  L

         410        420        430        440        450
GAGATCGTCG AAGTCCTGCT GAAGTACGGT GCCGACGTGA ACGCACTCGA
CTCTAGCAGC TTCAGGACGA CTTCATGCCA CGGCTGCACT TGCGTGAGCT
 E  I  V   E  V  L  L   K  Y  G   A  D  V   N  A  L  D

         460        470        480        490        500
CCTTATTGGT AAGACTCCAC TGCACCTGAC TGCTATTGAT GGCCATCTGG
GGAATAACCA TTCTGAGGTG ACGTGGACTG ACGATAACTA CCGGTAGACC
  L  I  G   K  T  P   L  H  L  T   A  I  D   G  H  L

         510        520        530        540        550
AGATCGTCGA AGTCCTGCTA AAGCACGGTG CGGACGTCAA TGCTCAGGAC
TCTAGCAGCT TCAGGACGAT TTCGTGCCAC GCCTGCAGTT ACGAGTCCTG
E  I  V  E   V  L  L   K  H  G  A  D  V  N   A  Q  D
```

```
       560        570        580        590        600
AAATTCGGTA AGACCGCTTT CGACATCTCC ATCGACAATG GTAACGAGGA
TTTAAGCCAT TCTGGCGAAA GCTGTAGAGG TAGCTGTTAC CATTGCTCCT
 K  F  G   K  T  A  F   D  I  S   I  D  N   G  N  E  D

       610        620        630        640        650
CCTGGCTGAA ATCCTGCAAA AGCTTAATGG CGCGCCTGGT TCTGGTGGTT
GGACCGACTT TAGGACGTTT TCGAATTACC GCGCGGACCA AGACCACCAA
  L  A  E   I  L  Q   K  L  N  G   A  P  G   S  G  G

       660        670        680        690        700
CTGGTGGTCC CTCGACATGC AGCAAGCCCA CGTGCCCACG TGAGTCCCAT
GACCACCAGG GAGCTGTACG TCGTTCGGGT GCACGGGTGC ACTCAGGGTA
S  G  G  P   S  T  C   S  K  P   T  C  P

       710        720        730        740        750
TAGCCTCACC CTCTAGCCCC AGCCCGGGGA GCCCCACCCC AGGGTGCCCC
ATCGGAGTGG GAGATCGGGG TCGGGCCCCT CGGGGTGGGG TCCCACGGGG

       760        770        780        790        800
CCAGGTGTTG ACTCTTCCCC GTCTCTCCCA CTGCAGCCCC TGAACTCCTG
GGTCCACAAC TGAGAAGGGG CAGAGAGGGT GACGTCGGGG ACTTGAGGAC
                                       P  P   E  L  L

       810        820        830        840        850
GGGGGACCGT CTGTCTTCAT CTTCCCCCCA AAACCCAAGG ACACCCTCAT
CCCCCTGGCA GACAGAAGTA GAAGGGGGGT TTTGGGTTCC TGTGGGAGTA
 G  G  P   S  V  F  I   F  P  P   K  P  K   D  T  L  M

       860        870        880        890        900
GATCTCACGC ACCCCCGAGG TCACATGCGT GGTGGTGGAC GTGAGCCAGG
CTAGAGTGCG TGGGGGCTCC AGTGTACGCA CCACCACCTG CACTCGGTCC
  I  S  R   T  P  E   V  T  C  V   V  V  D   V  S  Q

       910        920        930        940        950
ATGACCCCGA GGTGCAGTTC ACATGGTACA TAAACAACGA GCAGGTGCGC
TACTGGGGCT CCACGTCAAG TGTACCATGT ATTTGTTGCT CGTCCACGCG
D  D  P  E   V  Q  F   T  W  Y   I  N  N  E   Q  V  R

       960        970        980        990       1000
ACCGCCCGGC CGCCGCTACG GGAGCAGCAG TTCAACAGCA CGATCCGCGT
TGGCGGGCCG GCGGCGATGC CCTCGTCGTC AAGTTGTCGT GCTAGGCGCA
 T  A  R   P  P  L  R   E  Q  Q   F  N  S   T  I  R  V

      1010       1020       1030       1040       1050
GGTCAGCACC CTCCCCATCG CGCACCAGGA CTGGCTGAGG GGCAAGGAGT
CCAGTCGTGG GAGGGGTAGC GCGTGGTCCT GACCGACTCC CCGTTCCTCA
  V  S  T   L  P  I   A  H  Q  D   W  L  R   G  K  E

      1060       1070       1080       1090       1100
TCAAGTGCAA AGTCCACAAC AAGGCACTCC CGGCCCCCAT CGAGAAAACC
AGTTCACGTT TCAGGTGTTG TTCCGTGAGG GCCGGGGGTA GCTCTTTTGG
F  K  C  K   V  H  N   K  A  L   P  A  P  I   E  K  T

      1110       1120       1130       1140       1150
ATCTCCAAAG CCAGAGGTGG GAGCCGCGGG CTGGGAGCAG GGCAGGCAGC
```

```
TAGAGGTTTC GGTCTCCACC CTCGGCGCCC GACCCTCGTC CCGTCCGTCG
 I  S  K   A

      1160       1170       1180       1190       1200
TCCCACGGCC CGAGGCCTCC GCCCGGGAGT GACCCTGTGC TGTCCGCTGT
AGGGTGCCGG GCTCCGGAGG CGGGCCCTCA CTGGGACACG ACAGGCGACA

      1210       1220       1230       1240       1250
CCCCACAGGG CAGCCCCTGG AGCCGAAGGT CTACACCATG GGCCCTCCCC
GGGGTGTCCC GTCGGGGACC TCGGCTTCCA GATGTGGTAC CCGGGAGGGG
            Q  P  L   E  P  K  V   Y  T  M   G  P  P

      1260       1270       1280       1290       1300
GGGAGGAGCT GAGCAGCAGG TCGGTCAGCC TGACCTGCAT GATCAACGGC
CCCTCCTCGA CTCGTCGTCC AGCCAGTCGG ACTGGACGTA CTAGTTGCCG
R  E  E  L   S  S  R   S  V  S   L  T  C  M   I  N  G

      1310       1320       1330       1340       1350
TTCTACCCTT CCGACATCTC GGTGGAGTGG GAGAAGAACG GGAAGGCAGA
AAGATGGGAA GGCTGTAGAG CCACCTCACC CTCTTCTTGC CCTTCCGTCT
 F  Y  P   S  D  I  S   V  E  W   E  K  N   G  K  A  E

      1360       1370       1380       1390       1400
GGACAACTAC AAGACCACGC CGGCCGTGCT GGACAGCGAC GGCTCCTACT
CCTGTTGATG TTCTGGTGCG GCCGGCACGA CCTGTCGCTG CCGAGGATGA
  D  N  Y   K  T  T   P  A  V  L   D  S  D   G  S  Y

      1410       1420       1430       1440       1450
TCCTCTACAG CAAGCTCTCA GTGCCCACGA GTGAGTGGCA GCGGGGCGAC
AGGAGATGTC GTTCGAGAGT CACGGGTGCT CACTCACCGT CGCCCCGCTG
F  L  Y  S   K  L  S   V  P  T   S  E  W  Q   R  G  D

      1460       1470       1480       1490       1500
GTCTTCACCT GCTCCGTGAT GCACGAGGCC TTGCACAACC ACTACACGCA
CAGAAGTGGA CGAGGCACTA CGTGCTCCGG AACGTGTTGG TGATGTGCGT
 V  F  T   C  S  V  M   H  E  A   L  H  N   H  Y  T  Q

      1510       1520       1530       1540       1550
GAAGTCCATC TCCCGCTCTC CGGGTAAATG AGCGCTGTGC CGGCGAGCTG
CTTCAGGTAG AGGGCGAGAG GCCCATTTAC TCGCGACACG GCCGCTCGAC
  K  S  I   S  R  S   P  G  K  *
```
