# Supplementary material for: Utility of binding protein fusions to immunoglobulin heavy chain constant regions from mammalian and avian species
Source: J Biol Chem. 2025 Feb 18;301(4):108324. doi: 10.1016/j.jbc.2025.108324 (PMC11964738; doi:10.1016/j.jbc.2025.108324)
Supplement: Figure S6 [file mmc7.pdf]

Sequence: anti-GFP DARPin in Chicken Fc - Figure S6

```
                                                    50
GCGGCCGCCT GCACCTCGGT TCTATCGATT GAATTCCACC ATGGAGTGGG
CGCCGGCGGA CGTGGAGCCA AGATAGCTAA CTTAAGGTGG TACCTCACCC
                                             M  E  W

        60         70         80         90        100
GTTACCTGTT GGAAGTGACC TCGCTCCTAG CCGCCTTGGC GGTGCTACAG
CAATGGACAA CCTTCACTGG AGCGAGGATC GGCGGAACCG CCACGATGTC
G  Y  L  L   E  V  T   S  L  L   A  A  L  A   V  L  Q

       110        120        130        140        150
CGCTCTAGCG GCGCTGCCGC GGCTTCGGCC AAGGAGACGC GTGGTGTCGA
GCGAGATCGC CGCGACGGCG CCGAAGCCGG TTCCTCTGCG CACCACAGCT
 R  S  S   G  A  A  A   A  S  A   K  E  T   R  G  V  D

       160        170        180        190        200
CGGTGGTGAC CTGGGTAAGA AGCTGCTGGA AGCTGCTCGT GCTGGTCAGG
GCCACCACTG GACCCATTCT TCGACGACCT TCGACGAGCA CGACCAGTCC
  G  G  D   L  G  K   K  L  L  E   A  A  R   A  G  Q

       210        220        230        240        250
ACGACGAAGT TCGTATCCTG ATGGCTAACG GTGCCGATGT TAACGCACTT
TGCTGCTTCA AGCATAGGAC TACCGATTGC CACGGCTACA ATTGCGTGAA
D  D  E  V   R  I  L   M  A  N   G  A  D  V   N  A  L

       260        270        280        290        300
GACCGTTTTG GTCTTACTCC GCTGCACCTT GCTGCTCAGC GTGGCCACTT
CTGGCAAAAC CAGAATGAGG CGACGTGGAA CGACGAGTCG CACCGGTGAA
 D  R  F   G  L  T  P   L  H  L   A  A  Q   R  G  H  L

       310        320        330        340        350
AGAAATTGTT GAGGTTCTAC TGAAATGTGG TGCAGATGTA AATGCTGCTG
TCTTTAACAA CTCCAAGATG ACTTTACACC ACGTCTACAT TTACGACGAC
  E  I  V   E  V  L   L  K  C  G   A  D  V   N  A  A

       360        370        380        390        400
ACCTTTGGGG TCAGACTCCG CTGCACCTGG CTGCTACTGC TGGTCACTTA
TGGAAACCCC AGTCTGAGGC GACGTGGACC GACGATGACG ACCAGTGAAT
D  L  W  G   Q  T  P   L  H  L   A  A  T  A   G  H  L

       410        420        430        440        450
GAGATCGTCG AAGTCCTGCT GAAGTACGGT GCCGACGTGA ACGCACTCGA
CTCTAGCAGC TTCAGGACGA CTTCATGCCA CGGCTGCACT TGCGTGAGCT
 E  I  V   E  V  L  L   K  Y  G   A  D  V   N  A  L  D

       460        470        480        490        500
CCTTATTGGT AAGACTCCAC TGCACCTGAC TGCTATTGAT GGCCATCTGG
GGAATAACCA TTCTGAGGTG ACGTGGACTG ACGATAACTA CCGGTAGACC
  L  I  G   K  T  P   L  H  L  T   A  I  D   G  H  L

       510        520        530        540        550
AGATCGTCGA AGTCCTGCTA AAGCACGGTG CGGACGTCAA TGCTCAGGAC
TCTAGCAGCT TCAGGACGAT TTCGTGCCAC GCCTGCAGTT ACGAGTCCTG
E  I  V  E   V  L  L   K  H  G   A  D  V  N   A  Q  D
```

```
       560        570        580        590        600
AAATTCGGTA AGACCGCTTT CGACATCTCC ATCGACAATG GTAACGAGGA
TTTAAGCCAT TCTGGCGAAA GCTGTAGAGG TAGCTGTTAC CATTGCTCCT
 K  F  G   K  T  A  F   D  I  S   I  D  N   G  N  E  D

       610        620        630        640        650
CCTGGCTGAA ATCCTGCAAA AGCTTAATGG CGCGCCTGGT TCTGGTGGTT
GGACCGACTT TAGGACGTTT TCGAATTACC GCGCGGACCA AGACCACCAA
  L  A  E   I  L  Q   K  L  N  G   A  P  G   S  G  G

       660        670        680        690        700
CTGGTGACAA AACTCACACA TGCCCACCGT GCCCAGACGG CGCTCAGAGC
GACCACTGTT TTGAGTGTGT ACGGGTGGCA CGGGTCTGCC GCGAGTCTCG
S  G  D  K   T  H  T   C  P  P   C  P  D  G   A  Q  S

       710        720        730        740        750
TGCAGCCCCA TCCAGCTGTA CGCCATCCCA CCCAGCCCGG GCGAGCTGTA
ACGTCGGGGT AGGTCGACAT GCGGTAGGGT GGGTCGGGCC CGCTCGACAT
 C  S  P   I  Q  L  Y   A  I  P   P  S  P   G  E  L  Y

       760        770        780        790        800
CATCAGCTTA GACGCCAAAC TGAGGTGCCT GGTGGTCAAC CTGCCCAGCG
GTAGTCGAAT CTGCGGTTTG ACTCCACGGA CCACCAGTTG GACGGGTCGC
  I  S  L   D  A  K   L  R  C  L   V  V  N   L  P  S

       810        820        830        840        850
ATTCCAGCCT CAGCGTCACC TGGACCAGGG AGAAGAGTGG GAACCTCCGG
TAAGGTCGGA GTCGCAGTGG ACCTGGTCCC TCTTCTCACC CTTGGAGGCC
D  S  S  L   S  V  T   W  T  R   E  K  S  G   N  L  R

       860        870        880        890        900
CCCGACCCGA TGGTCCTCCA AGAACACTTC AACGGCACCT ACAGCGCCAG
GGGCTGGGCT ACCAGGAGGT TCTTGTGAAG TTGCCGTGGA TGTCGCGGTC
 P  D  P   M  V  L  Q   E  H  F   N  G  T   Y  S  A  S

       910        920        930        940        950
CAGCGCCGTC CCCGTCAGCA CCCAGGATTG GTTATCCGGG GAGAGGTTCA
GTCGCGGCAG GGGCAGTCGT GGGTCCTAAC CAATAGGCCC CTCTCCAAGT
  S  A  V   P  V  S   T  Q  D  W   L  S  G   E  R  F

       960        970        980        990       1000
CCTGCACCGT GCAGCACGAG GAGCTGCCCC TGCCGCTCAG CAAGAGCGTC
GGACGTGGCA CGTCGTGCTC CTCGACGGGG ACGGCGAGTC GTTCTCGCAG
T  C  T  V   Q  H  E   E  L  P   L  P  L  S   K  S  V

      1010       1020       1030       1040       1050
TACAGGAACA CGGGACCCAC CACCCCACCT CTGATCTACC CCTTCGCCCC
ATGTCCTTGT GCCCTGGGTG GTGGGGTGGA GACTAGATGG GGAAGCGGGG
 Y  R  N   T  G  P  T   T  P  P   L  I  Y   P  F  A  P

      1060       1070       1080       1090       1100
CCACCCGGAA GAGCTGTCCC TCTCCCGCGT CACCTTGAGC TGCCTGGTCC
GGTGGGCCTT CTCGACAGGG AGAGGGCGCA GTGGAACTCG ACGGACCAGG
  H  P  E   E  L  S   L  S  R  V   T  L  S   C  L  V

      1110       1120       1130       1140       1150
GCGGCTTCCG CCCACGTGAC ATCGAGATCC GGTGGCTCCG CGACCACCGC
```

```
CGCCGAAGGC GGGTGCACTG TAGCTCTAGG CCACCGAGGC GCTGGTGGCG
R  G  F  R   P  R  D   I  E  I   R  W  L  R   D  H  R

      1160       1170       1180       1190       1200
GCCGTTCCCG CCACCGAATT CGTCACCACC GCCGTCCTAC CGGAAGAGAG
CGGCAAGGGC GGTGGCTTAA GCAGTGGTGG CGGCAGGATG GCCTTCTCTC
 A  V  P   A  T  E  F   V  T  T   A  V  L   P  E  E  R

      1210       1220       1230       1240       1250
AACCGCAAAC GGCGCCGGCG GTGACGGCGA CACCTTCTTC GTGTACAGTA
TTGGCGTTTG CCGCGGCCGC CACTGCCGCT GTGGAAGAAG CACATGTCAT
  T  A  N   G  A  G   G  D  G  D   T  F  F   V  Y  S

      1260       1270       1280       1290       1300
AGATGAGCGT GGAGACCGCC AAGTGGAACG GCGGGACGGT GTTCGCCTGC
TCTACTCGCA CCTCTGGCGG TTCACCTTGC CGCCCTGCCA CAAGCGGACG
K  M  S  V   E  T  A   K  W  N   G  G  T  V   F  A  C

      1310       1320       1330       1340       1350
ATGGCGGTGC ACGAGGCGCT GCCCATGCGC TTCAGCCAGC GCACGCTGCA
TACCGCCACG TGCTCCGCGA CGGGTACGCG AAGTCGGTCG CGTGCGACGT
 M  A  V   H  E  A  L   P  M  R   F  S  Q   R  T  L  Q

      1360       1370       1380       1390       1400
GAAACAGGCT GGTAAAGGTG GTTCTGGTGG TCATCACCAC CATCATCATC
CTTTGTCCGA CCATTTCCAC CAAGACCACC AGTAGTGGTG GTAGTAGTAG
  K  Q  A   G  K  G   G  S  G  G   H  H  H   H  H  H

      1410       1420       1430
ACCACTGAGG GCCCGAGCTT GGCCGCCATG GCCCAACT
TGGTGACTCC CGGGCTCGAA CCGGCGGTAC CGGGTTGA
H  H  *
```
